# Impact of the COVID-19 pandemic on quality of tuberculosis care in private facilities in Bandung, Indonesia: a repeated cross-sectional standardized patients study

**DOI:** 10.1186/s12889-023-17001-y

**Published:** 2024-01-05

**Authors:** Angelina Sassi, Bony Wiem Lestari, Kuuni Ulfah Naila El Muna, Charity Oga-Omenka, Nur Afifah, Rodiah Widarna, Lavanya Huria, Nathaly Aguilera Vasquez, Andrea Benedetti, Panji Fortuna Hadisoemarto, Benjamin Daniels, Jishnu Das, Madhukar Pai, Bachti Alisjahbana

**Affiliations:** 1 Department of Epidemiology, Biostatistics, and Occupational Health, and McGill International TB Centre, McGill University, Montreal, Canada; 2 Research Center for Care and Control of Infectious Disease, Universitas Padjadjaran, Bandung, Indonesia; 3 Department of Public Health, Universitas Padjadjaran, Bandung, Indonesia; 4 Department of Public Health, Universitas Nahdlatul Ulama Surabaya, Surabaya, Indonesia; 5 School of Public Health Sciences, University of Waterloo, Waterloo, Canada; 6 Department of Medicine, McGill University, Montreal, Canada; 7 McCourt School of Public Policy, Georgetown University, Washington, DC, USA; 8 Department of Internal Medicine, Dr. Hasan Sadikin General Hospital, Bandung, Indonesia

**Keywords:** Tuberculosis, COVID-19, Quality of care, Indonesia, Private sector

## Abstract

**Background:**

Indonesia has the second highest incidence of tuberculosis in the world. While 74% of people with tuberculosis in Indonesia first accessed the private health sector when seeking care for their symptoms, only 18% of tuberculosis notifications originate in the private sector. Little is known about the impact of the COVID-19 pandemic on the private sector. Using unannounced standardized patient visits to private providers, we aimed to measure quality of tuberculosis care during the COVID-19 pandemic.

**Methods:**

A cross-sectional study was conducted using standardized patients in Bandung City, West Java, Indonesia. Ten standardized patients completed 292 visits with private providers between 9 July 2021 and 21 January 2022, wherein standardized patients presented a presumptive tuberculosis case. Results were compared to standardized patients surveys conducted in the same geographical area before the onset of COVID-19.

**Results:**

Overall, 35% (95% confidence interval (CI): 29.2–40.4%) of visits were managed correctly according to national tuberculosis guidelines. There were no significant differences in the clinical management of presumptive tuberculosis patients before and during the COVID-19 pandemic, apart from an increase in temperature checks (adjusted odds ratio (aOR): 8.05, 95% CI: 2.96–21.9, *p* < 0.001) and a decrease in throat examinations (aOR 0.16, 95% CI: 0.06–0.41, *p* = 0.002) conducted during the pandemic.

**Conclusions:**

Results indicate that providers successfully identify tuberculosis in their patients yet do not manage them according to national guidelines. There were no major changes found in quality of tuberculosis care due to the COVID-19 pandemic. As tuberculosis notifications have declined in Indonesia due to the COVID-19 pandemic, there remains an urgent need to increase private provider engagement in Indonesia and improve quality of care.

**Supplementary Information:**

The online version contains supplementary material available at 10.1186/s12889-023-17001-y.

## Background

Globally, COVID-19-related disruptions have resulted in two million more people left undiagnosed or unreported with tuberculosis (TB) across 2020 and 2021 compared to 2019. Thus, the number of people with undiagnosed and untreated TB has increased, resulting in more TB deaths and infections. Indonesia, a country with the second highest incidence of TB in the world with close to one million new cases per year, experienced the second highest reduction in case notifications between 2019 and 2020 among all high TB burden countries [1]. Indonesia is also is one of the four countries that accounted for most of the estimated increase in TB deaths globally in 2021 [1].

The COVID-19-related disruptions to TB care happened due to several factors. Health service availability for people with TB symptoms was drastically reduced in Indonesia, especially during the Delta wave, due to stay-at-home directives, diversion of resources, facility closures, and provider deaths [2–7]. TB care-seeking was also reduced due to stay-at-home directives and fears of contracting COVID-19 at healthcare facilities [3, 5]. The similarity of symptoms across TB and COVID-19 could have also affected the quality of TB services in the country, as this may lead to diagnostic confusion [8]. Recognizing this issue, the President of Indonesia encouraged facilities to implement bi-directional screening for TB and COVID-19 [9].

Private healthcare sectors globally were also affected by the COVID-19 pandemic [10, 11]. Indonesia has a large private health sector, accounting for 63% of outpatient healthcare utilization and 74% of initial visits for TB care [12]. Even before the pandemic, the sector had limited engagement with the Indonesia National Tuberculosis Programme (NTP): an analysis of national TB data from 2018 showed that despite the high preference among Indonesians towards the private sector, the private sector accounts for only 18% of TB notifications [13]. Furthermore, the quality of care in the private sector is concerning: a multicenter study showed that most private general practitioners (GPs) were not aware of the International Standard of Tuberculosis Care (ISTC) and had not undertaken any related training. Inaccurate prescription was also evident, with the rate of second-line prescription reaching as high as 45.5% in one city [14]. To strengthen the private sector and its involvement with the NTP, the Ministry of Health of Indonesia and representatives from 13 professional organizations began a district public private mix (DPPM) project by first establishing a coalition of professional organizations against TB, called KOPI-TB, beginning in 2018. The member organizations of KOPI-TB are mandated to disseminate the updated TB management to their members (physicians, pharmacists, etc.). One of the main DPPM initiatives is Coach TB, which aims to improve the capacity of health workers and improve the quality of tuberculosis services in the private health sector [15]; however, the rollout of this project and other planned DPPM implementation activities had not begun by 2020 [16]. The COVID-19 pandemic introduces additional urgency surrounding efforts to understand these existing and widening gaps in the quality of TB screening and diagnosis in the private sector.

The use of standardized patients (SPs), or individuals recruited from the local community to present the same case to multiple providers in a blinded fashion, is considered the gold standard for measurement of clinical correctness of care, an important aspect of healthcare quality [17]. Since SPs present the same case to each provider, confounders related to differential patient and case-mix are better controlled than in other approaches to ascertain quality of care such as administrative data or medical records [18]. Additionally, SP studies allow researchers to take accurate measurements of multidimensional quality outcomes with no missing observations and without the Hawthorne effect, wherein providers change their behavior when they know they are being observed [19]. Previous SP studies of private provider management in four high TB-burden countries have found that between 21 and 43% of SPs presenting with TB symptoms were offered appropriate diagnostic tests, and many were offered broad-spectrum antibiotics and steroids, which can mask TB symptoms and increase the risk of antibiotic resistance [19–23].

Few studies to date have investigated how the quality of TB services was impacted by the COVID-19 pandemic [24–26]. A pre-pandemic SP study called Investigation of services delivered for TB by external care system – especially the private sector (INSTEP) was conducted in Bandung, Indonesia in 2018–2019. This study found that 32% of private GPs and 19% of private specialists correctly managed SPs presenting with presumptive TB symptoms according to Indonesian national guidelines, compared to 87% of providers at public community health centers (CHC, Indonesian: *Pusat Kesehatan Masyarakat* [*Puskesmas*]) [27]. The COVID Effects on TB Services in the Private Sector (COVET) study set out to evaluate the impact of the COVID-19 pandemic on tuberculosis services in the private health care sectors of India, Indonesia, and Nigeria. As part of this study, we sought to understand whether private providers in Indonesia are correctly managing patients with TB symptoms, as defined by national guidelines. Additionally, we aimed to investigate which types of private providers are more likely to correctly manage patients with TB symptoms, and to estimate the extent of changes in clinical practices in TB care during the COVID-19 pandemic by comparing with results from the INSTEP SP study.

## Methods

### Study setting

A cross-sectional study was conducted between 9 July 2021 and 21 January 2022 using standardized patients (SPs) in Bandung City, West Java, Indonesia. Bandung is the capital of West Java Province and the fourth most populous city in Indonesia, with a population of 2.4 million [28]. Bandung is divided into 30 administrative districts with 32 hospitals, 80 Puskesmas, 16 public clinics, and 342 private clinics. In 2019, there were 3,623 registered general practitioners and 955 specialists in West Java province across the public and private health sectors [29]. Dual practice is common in Indonesia, with reports of up to 70% of Puskesmas general practitioners and virtually all public health sector specialists engaging in private practice [12, 30]

### Sampling frame and sample size

The sampling frame for this study matched a pre-COVID-19 SP study conducted in 2018–2019 (INSTEP) [27]. The INSTEP SP study examined TB management among public and private sector providers and the presumptive TB case identical to the one used in this study, and three other cases in which SPs presented as patients who had already received sputum test results. The presumptive TB case scenario used in this study is identical to the one used in 67 standardized patient visits made to private providers in the INSTEP SP study.

For both studies, 36 Puskesmas were randomly selected from the 80 Puskesmas in Bandung. A mapping survey of all private practitioners in these catchment areas was conducted from 15 to 2021 to 27 December 2021. Eligible participants were all private practitioners who participated in the mapping survey and who indicated that they were currently providing health services for patients with general symptoms, including fever, cough, and shortness of breath.

We anticipated that 30% of the SP visits will be managed correctly, based on the INSTEP SP survey in Bandung. A sample size of 275 interactions would allow us to estimate this proportion with a 95% confidence interval of 20–41%. The comparison between SP visits in the INSTEP study and this study used fixed sample sizes as we had no control over the sample size in the INSTEP study. A post-hoc power calculation indicates that with 67 SP interactions in the pre-pandemic survey, and 275 interactions in the present study, we had a power of 80% to detect a drop of 15% points in the correct management proportion.

### SP training and case scenario

Ten SPs were recruited from Bandung residents, seven of whom had participated in the INSTEP SP study. SPs were determined healthy after being screened for TB and COVID-19, and were then trained using the clinical scenario, exit questionnaire and standard operational procedure adapted from Kwan et al. [18].

SPs presented a presumptive TB case, telling the doctor that they have had a cough for 2–3 weeks. If prompted, SPs would disclose that they also have a productive cough with yellow phlegm and without blood, an intermittent mid-grade fever, night sweats, loss of appetite, weight loss, fatigue, and that they self-medicated for 1 week using only acetaminophen and cough syrup with no improvement. These details are identical to the presumptive TB case presentation in the INSTEP SP study. Where the COVET case differed from the INSTEP study was in the additional standardization of SP responses to questions about COVID-19 (Table 1 and Supplement 1).

**Table 1:** Description of clinical case scenario

**Case description**	**Patient presentation**	**Expected correct management based on 2016 NTP guidelines**	**Expected correct management based on prior SP TB studies**
A classic case of suspected tuberculosis with a cough for 2–3 weeks and a low-grade fever, cold sweats, loss of appetite and other typical symptoms of TB.	A suspected case of tuberculosis was conveyed to a doctor at a private health service by saying: “Doctor, I have a cough that doesn’t get better.” The patient has no history of COVID-19, has never been tested for COVID-19, but has received a first dose of a COVID-19 vaccine.	Recommendations: 1. Ordered for Xpert MTB/RIF test or AFB smear 2. Referred to Public DOTS center	Recommendations: 1. Ordered for Chest X-rays and/or sputum test (AFB smear, X-pert MTB/RIF, Culture, DST) 2. Referral to another provider or public TB services.

### SP visits and data collection

**Fig. 1:**
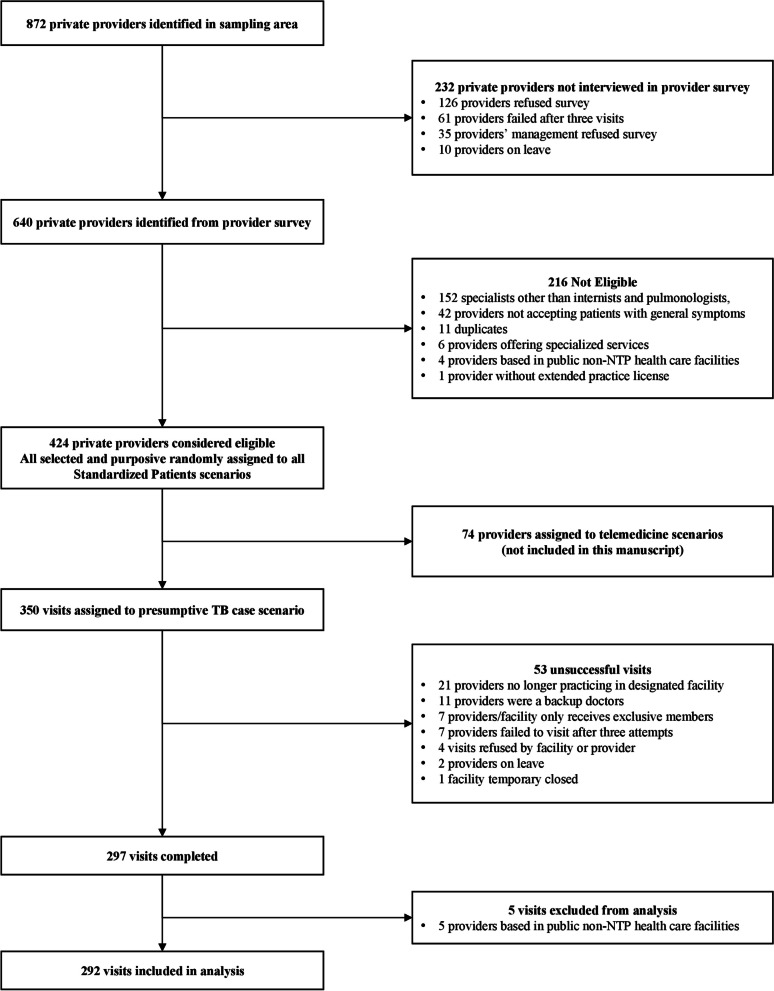
Study sampling

The mapping survey described above resulted in a total of 424 doctors eligible to be visited in this study, of whom 74 providers were excluded as they were assigned virtual SP visits as part of a pilot study on whether the SP methodology could be used in telehealth consultations (Fig. 1). All remaining 350 providers were assigned to the presumptive TB case scenario. SPs were purposively assigned to providers to avoid detection as an SP or as a patient with relapsed TB. Additionally, all female SPs were assigned to female doctors to ensure their comfort and safety.

We conducted two rounds of piloting to ensure that the scenario and exit questionnaire were coherent and reasonable. After piloting, each doctor received an unannounced visit from one SP during business hours. All SPs paid the providers their usual fee and paid for and collected medicines up to a budget limit of 300,000 Indonesian rupiah (IDR) (USD $20) for general practitioner visits and 350,000 IDR (USD $23) for specialist visits. If the total amount of the medicines and visit exceeded the budget, SPs were trained to retain all prescriptions but only redeem half of the medicine.

Data were collected by SPs using a voice recorder and were documented using an exit questionnaire immediately after the visit. The exit questionnaire, referral slips, collected medicines, and any unfilled prescriptions were later checked by our research team before being entered into an electronic based data hosted at Universitas Padjadjaran [Research Electronic Data Capture (REDCap)] [31].

### COVID-19 context and adaptations

**Fig. 2:**
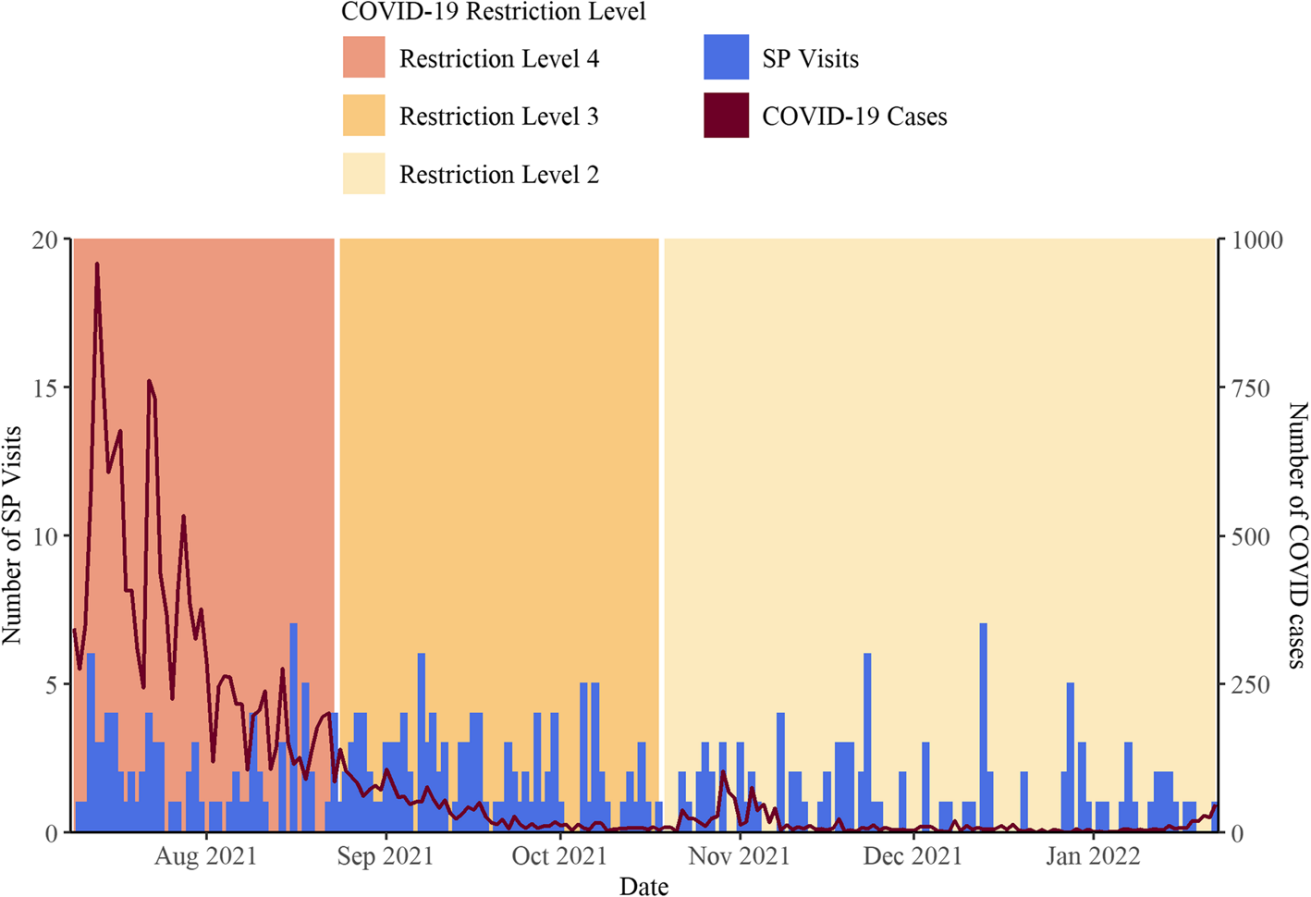
Timeline of SP visits and COVID-19 statistics in Bandung City, West Java, Indonesia. Legend: Restriction Level 4 was implemented when there were more than 100 cases per 100,000 population per week, and included supermarkets open at 50% capacity, non-essential sector working entirely from home, shopping centers and malls closed, restaurants open for take-away only, and places of worship closed. Restriction Level 3 was implemented when weekly confirmed cases were between 65 and 100 cases per 100,000 population, and included supermarkets open at 50% capacity, shopping centers and malls open at 25% capacity, restaurants open for dining with 25% capacity, and places of worship closed. Restriction Level 2 was implemented when weekly confirmed cases were between 40 and 64 per 100,000 population, and included supermarkets open at 75% capacity, shopping centers and malls open at 50% capacity, restaurants open for dining with 50% capacity, places of worship open with 50% capacity and strict procedures, and public facilities open with 50% capacity and strict procedures. Acronyms: SP = Standardized Patient

Figure 2 shows the alignment between COVID-19 case numbers in Bandung City against the dates of the SP visits conducted in this study. SP visits were conducted amidst three different COVID-19 restriction levels in Bandung (Fig. 2).

At the time of data collection, we required SPs to be vaccinated with two doses of a COVID-19 vaccine. SPs were directed to wear masks during each visit and follow all other COVID-19 prevention directives as indicated by the facilities (physical distancing and handwashing). If any SPs developed symptoms of respiratory illness, they were directed to be tested at the nearest Puskesmas.

The study team was required to make some adaptations to the methodology due to COVID-19 related public health restrictions. When visits began, the field team discovered that some doctors would refuse patients who had not been vaccinated. To mitigate this, on 26 July 2021 we changed the protocol to allow SPs to disclose to the provider that they have had one dose of a COVID-19 vaccine. Additionally, four providers visited in August and September 2021 asked patients to get tested for COVID-19 at the facility before consultation, so the decision was made in August 2021 to allow SPs to submit for this test to allow SP visits to continue.

### Outcome definition

The main outcome of this study is correct management, judged using concordance with the 2016 NTP guidelines as the benchmark for management of presumptive tuberculosis [32]. These guidelines stipulate that correct management of presumptive tuberculosis for new patients with no history of prior TB treatment, no history of close contact with rifampicin-resistant TB patients, and unknown HIV status or HIV-negative patients is clinical assessment and bacteriological examination by sputum smear or rapid molecular test (GeneXpert MTB/Rif (Xpert), Cepheid, Sunnyvale, CA). Referral to directly observed therapy shortcourse (DOTS) center was determined as correct management under these guidelines (Table 1). These guidelines were updated in 2021 to stipulate that all those with presumptive tuberculosis should be examined with Xpert [33]. While we present data on the use of Xpert as well as the percentage of cases that were correctly managed according to the 2021 guidelines, for our main outcome we chose to use the 2016 guidelines. This is primarily to compare the latest findings with the INSTEP SP study, and as implementation of the 2021 guideline has been slowed in part due to the COVID-19 pandemic.

Finally, the algorithm for expected correct management used in prior SP TB studies was used as a secondary benchmark in order to compare these results to previous SP studies conducted in other contexts [20]. These guidelines stipulate that correct management of presumptive tuberculosis is recommendation for chest X-ray and/or any sputum test (Acid-Fast Bacilli (AFB) smear, Xpert, Culture, Drug susceptibility testing (DST)), or referral to another provider or public TB services. These outcome measures take a lenient approach in which providers were not penalized for the use of unnecessary or even potentially harmful medicines, and thus the results presented are upper-bound estimates of clinical correctness, as measured by adherence to TB standards of care.

### Comparison with pre-COVID-19 SP study

As this SP study was conducted in the same geographical area as the INSTEP SP study conducted in 2018–2019, we first compared characteristics of the SP visits before and during the COVID-19 pandemic. To determine which SP visits were conducted with the same providers in both studies, we manually cross-checked the names, health facilities, and age of doctors in both studies and created a unique ID system spanning the two studies. Comparisons were made between the 67 standardized patient visits made to the private providers in the INSTEP SP study using the presumptive TB case scenario identical to the one used in this study (292 visits).

### Data analysis

Descriptive analysis evaluated proportions and 95% confidence intervals (CI) of provider and facility characteristics captured in the provider mapping survey (doctor’s qualification, age, sex, and whether they have attended TB management training [collected during the provider mapping exercise that formed the sampling frame for the SP study]; type of facility, facility linkage to NTP, whether the facility accepts the national insurance [*Jaminan Kesehatan Nasional* (JKN)], availability of chest X-rays and sputum test in the facility), duration of the SP visit, clinic patient density measured by number of other patients waiting before and after the visit, medical history taken by staff or doctor, health education, diagnosis, referral, medication prescribed by doctor, total cost of the visit, and concordance to NTP guidelines.

Generalized linear mixed models fit with quadrature were used to understand which private provider and facility characteristics are associated with correct management of patients with TB symptoms (R version 4.1.0/R Studio version 2022.07.1). Random intercepts were included for each SP (to account for the sampling structure which only randomized female SPs to visit female providers) and for each facility (to account for probable clustering by facility, wherein providers from the same facility would be more like each other). Adjusted odds ratios (aOR) with 95% CI were reported. Confounding variables were chosen using a directed acyclic graph (DAG) created based on expert opinion. Further details on confounder selection are included in Supplement 2.

In the direct comparison between the INSTEP and COVET SP datasets, as fewer provider and facility characteristic variables were available in the INSTEP SP dataset compared to the COVET SP dataset due to changes in the mapping survey, only provider qualification and provider age were included as confounders in all models. The exception to this was that whether the provider had prescribed any medication was included as a confounder in the model describing visit cost by study year, as prescriptions strongly influence the total visit cost. The Bonferroni-Holm multiple test procedure was used to account for multiple comparisons made among visit characteristics.

## Results

### Characteristics of the SP visits

A total of 297 visits were completed by ten SPs between 9 July 2021 and 21 January 2022, out of 350 visits attempted, resulting in an overall completion rate of 85%. Five visits completed at public non-NTP facilities were excluded from our analysis, resulting in a total sample of 292 visits (Fig. 1). Each SP completed an average of 29 visits (Standard Deviation (SD): 3.01) which lasted 9.7 min on average (SD: 6.5). SPs paid on average IDR 121,869 (SD: IDR 75,417) [USD $8.40 (SD: $5.20)] for the total cost of the visit. One third of visits were conducted under COVID-19 Restriction Level 2 (34%), 39% under Restriction Level 3, and 27% under Restriction Level 4.

#### Provider and facility demographics

The SPs visited 292 providers practicing at 165 facilities (Table 2). Most facilities were clinics or hospitals with more than one provider (70%), not linked to the Indonesian NTP (98%), had a pharmacy attached to the facility (81%), and 37% accepted Indonesian national insurance (JKN). The providers were on average 40 years old, 92% were general practitioners, and 61% were female. When inquired during the mapping survey, 61% of the providers indicated they had previously received TB management training and 57% diagnosed at least one TB case each month.

**Table 2:** Provider and facility characteristics

**Provider characteristics**	**INSTEP study, 2018–2019, ** ***N*** ** = 67**^**a**^	**COVET study, 2021–2022, *N* = 292**^**a**^
Provider consented to mapping survey	56 (84%)	289 (99%)
Provider sex		
Female	37 (55%)	177 (61%)
Male	30 (45%)	115 (39%)
Provider age^b^	45 (15)	39 (12)
Provider qualification		
General Practitioner	52 (78%)	269 (92%)
Specialist	15 (22%)	23 (7.9%)
Internist	14 (93%)	18 (78%)
Pulmonologist	1 (6.7%)	5 (22%)
Provider diagnoses at least one TB patient per month^c^	33 (59%)	165 (57%)
Average number of TB patients diagnosed per month^c^		
0	23 (41%)	124 (43%)
< 1^d^	14 (25%)	0 (0%)
1–4	16 (29%)	129 (45%)
5 or more	3 (5.4%)	36 (12%)
Provider dispenses TB treatment to TB patients^c^	12 (21%)	74 (26%)
Provider has received TB management training^c,e^	**---**	175 (61%)
**Facility characteristics**	**INSTEP study, 2018–2019, *N* = 56**^**a**^	**COVET study, 2021–2022, *N* = 165**^**a**^
Facility type		
Clinic/hospital	44 (79%)	116 (70%)
Solo practice	12 (21%)	49 (30%)
Health sector & linkage to NTP		
Private HCF linked to NTP	1 (1.8%)	4 (2.4%)
Private HCF not linked to NTP	55 (98%)	161 (98%)
Additional services present at facility^c^		
Pharmacy	39 (70%)	131 (81%)
Laboratory	12 (21%)	34 (21%)
X-ray	6 (11%)	10 (6.2%)
Sputum examination^e^	---	8 (4.9%)
In-patient beds^e^	---	11 (6.8%)
Facility collaborates with Indonesian national health insurance (JKN)^c^	13 (23%)	59 (37%)

Acronyms: *INSTEP *Investigation of services delivered for TB by external care system - especially the private sector, *COVET  *COVID Effects on TB Services in the Private Sector, *TB *Tuberculosis, *NTP *National Tuberculosis Programme, *HCF *Health care facility, *JKN Jaminan Kesehatan Nasional*

^a^n(%); Mean (SD)

^b^Missing age from 13 providers in INSTEP and 6 providers in COVET

^c^Missing information from providers and facilities who declined the mapping survey

^d^Option not included in COVET study

^e^Not asked in INSTEP study

#### Provider screening behaviors

Detailed information about the SP visits can be found in Table 3. On average, providers asked 15 history-taking questions (SD: 6.4) and conducted five physical examinations (SD: 1.87) per visit. On average, providers asked SPs about three (SD: 1.12) out of five cardinal TB symptoms (cough duration, blood in sputum, fever, night sweats, and weight loss) with 13% asking about all five symptoms. The most asked questions in the history-taking portion of the visit were cough duration (99%), fever (90%), and whether the SP had taken any medications for their current symptoms (70%).

**Table 3:** Descriptive comparison of characteristics of the SP visits in INSTEP and COVET studies

**Characteristic**	**INSTEP study, 2018–2019**		**COVET study, 2021–2022**		**Difference between studies**			
	***N*** = 67^**a**^	**95% CI**^**b**^	***N* = 292**^**a**^	**95% CI**^**b**^	**Difference**^**c**^	**95% CI**^**c,b**^	***p*-value**^**c**^	**q-value**^**d**^
**Basic Visit Information**								
Time spent with provider (min)	10.9 (7.6)	9.0, 13	9.7 (6.5)	9.0, 10	-1.2	-3.2, 0.84	0.3	> 0.9
Number of patients when SP arrived at facility	3.1 (3.9)	2.1, 4.1	3.7 (6.2)	3.0, 4.4	0.62	-0.57, 1.8	0.3	> 0.9
Number of patients when SP left facility	2.3 (2.7)	1.6, 2.9	2.7 (4.4)	2.2, 3.2	0.44	-0.38, 1.3	0.3	> 0.9
**Correct Management (outcome)**								
Visit managed in concordance to NTP 2016 guidelines	19 (28%)	18%, 41%	101 (35%)	29%, 40%	6.2%	-6.8%, 19%	0.4	> 0.9
Visit managed in concordance to NTP 2021 guidelines	0 (0%)	0.00%, 6.8%	3 (1.0%)	0.27%, 3.2%	1.0%	-1.0%, 3.1%	> 0.9	> 0.9
Visit managed correctly based on prior SP studies	48 (72%)	59%, 82%	236 (81%)	76%, 85%	9.2%	-3.4%, 22%	0.13	> 0.9
**Symptoms and History-Taking**								
Number of history-taking questions asked	11.0 (4.3)	9.9, 12	10.5 (4.2)	10.0, 11	-0.53	-1.7, 0.62	0.4	> 0.9
Number of cardinal TB symptoms asked in history-taking (cough duration, blood in sputum, fever, night sweats, weight loss)	3.39 (1.13)	3.1, 3.7	3.26 (1.12)	3.1, 3.4	-0.13	-0.43, 0.17	0.4	> 0.9
Provider asked about cough duration	64 (96%)	87%, 99%	289 (99%)	97%, 100%	3.5%	-2.6%, 9.5%	0.14	> 0.9
Provider asked about cough with sputum	59 (88%)	77%, 94%	244 (84%)	79%, 88%	-4.5%	-14%, 5.3%	0.5	> 0.9
Provider asked about sputum color	33 (49%)	37%, 62%	121 (41%)	36%, 47%	-7.8%	-22%, 6.3%	0.3	> 0.9
Provider asked about blood in sputum	21 (31%)	21%, 44%	69 (24%)	19%, 29%	-7.7%	-21%, 5.3%	0.2	> 0.9
Provider asked about cough intensity (number of times per day)	25 (37%)	26%, 50%	122 (42%)	36%, 48%	4.5%	-9.3%, 18%	0.6	> 0.9
Provider asked about fever	57 (85%)	74%, 92%	264 (90%)	86%, 93%	5.3%	-4.8%, 15%	0.3	> 0.9
Provider asked about fever type (severe, moderate, mild)	29 (43%)	31%, 56%	94 (32%)	27%, 38%	-11%	-25%, 2.8%	0.11	> 0.9
Provider asked about appetite	28 (42%)	30%, 54%	130 (45%)	39%, 50%	2.7%	-11%, 17%	0.8	> 0.9
Provider asked about weight loss	39 (58%)	46%, 70%	175 (60%)	54%, 66%	1.7%	-12%, 16%	> 0.9	> 0.9
Provider asked about night sweats	46 (69%)	56%, 79%	154 (53%)	47%, 59%	-16%	-29%, -2.5%	0.026	> 0.9
Provider asked about wheezing	19 (28%)	18%, 41%	55 (19%)	15%, 24%	-9.5%	-22%, 3.1%	0.12	> 0.9
Provider asked about shortness of breath/difficulty breathing	33 (49%)	37%, 62%	182 (62%)	56%, 68%	13%	-1.0%, 27%	0.067	> 0.9
Provider asked about chest pain	23 (34%)	23%, 47%	73 (25%)	20%, 30%	-9.3%	-23%, 4.0%	0.2	> 0.9
Provider asked if SP had taken any medications for current symptoms	43 (64%)	51%, 75%	203 (70%)	64%, 75%	5.3%	-8.2%, 19%	0.5	> 0.9
Provider asked the name of the medications	38 (57%)	44%, 69%	174 (60%)	54%, 65%	2.9%	-11%, 17%	0.8	> 0.9
Provider asked how long the SP had taken the medications	12 (18%)	10.0%, 30%	47 (16%)	12%, 21%	-1.8%	-13%, 9.2%	0.9	> 0.9
Provider asked if SP had taken any antibiotics for current symptoms	2 (3.0%)	0.52%, 11%	46 (16%)	12%, 21%	13%	6.0%, 20%	0.010	0.9
Provider asked if SP takes long-term medications for other diseases	0 (0%)	0.00%, 6.8%	9 (3.1%)	1.5%, 6.0%	3.1%	0.18%, 6.0%	0.3	> 0.9
Provider asked about drug allergies	40 (60%)	47%, 71%	177 (61%)	55%, 66%	0.91%	-13%, 15%	> 0.9	> 0.9
Provider asked SP’s family/close contacts have similar symptoms	17 (25%)	16%, 38%	114 (39%)	33%, 45%	14%	0.92%, 26%	0.051	> 0.9
Provider asked about SP’s TB history	4 (6.0%)	1.9%, 15%	28 (9.6%)	6.6%, 14%	3.6%	-3.9%, 11%	0.5	> 0.9
Provider asked about family history of TB	14 (21%)	12%, 33%	66 (23%)	18%, 28%	1.7%	-10%, 13%	0.9	> 0.9
Provider asked if SP lives at home with children	4 (6.0%)	1.9%, 15%	12 (4.1%)	2.2%, 7.3%	-1.9%	-8.9%, 5.2%	0.7	> 0.9
Provider asked about anti-TB drug history	1 (1.5%)	0.08%, 9.1%	9 (3.1%)	1.5%, 6.0%	1.6%	-2.8%, 6.0%	0.8	> 0.9
Provider asked about history of diabetes	0 (0%)	0.00%, 6.8%	26 (8.9%)	6.0%, 13%	8.9%	4.7%, 13%	0.023	> 0.9
Provider asked about history of HIV	0 (0%)	0.00%, 6.8%	2 (0.7%)	0.12%, 2.7%	0.68%	-0.95%, 2.3%	> 0.9	> 0.9
Provider asked about history of hypertension	3 (4.5%)	1.2%, 13%	33 (11%)	8.0%, 16%	6.8%	-0.23%, 14%	0.15	> 0.9
Provider asked about other disease history	8 (12%)	5.7%, 23%	35 (12%)	8.6%, 16%	0.05%	-8.6%, 8.7%	> 0.9	> 0.9
Provider asked about alcohol consumption	0 (0%)	0.00%, 6.8%	2 (0.7%)	0.12%, 2.7%	0.68%	-0.95%, 2.3%	> 0.9	> 0.9
Provider asked about smoking status	29 (43%)	31%, 56%	93 (32%)	27%, 38%	-11%	-25%, 2.5%	0.10	> 0.9
Provider seemed to take note of the information provided by SP	59 (88%)	77%, 94%	260 (89%)	85%, 92%	1.0%	-8.5%, 10%	> 0.9	> 0.9
**Physical Examinations Conducted**								
Number of exams conducted	3.75 (1.74)	3.3, 4.2	3.77 (1.54)	3.6, 3.9	0.02	-0.43, 0.48	> 0.9	> 0.9
Provider measured pulse rate	46 (69%)	56%, 79%	218 (75%)	69%, 79%	6.0%	-7.1%, 19%	0.4	> 0.9
Provider measured temperature with thermometer	17 (25%)	16%, 38%	193 (66%)	60%, 71%	41%	28%, 53%	< 0.001	< 0.001
Provider measured blood pressure	56 (84%)	72%, 91%	254 (87%)	82%, 91%	3.4%	-7.2%, 14%	0.6	> 0.9
Provider conducted throat examination	37 (55%)	43%, 67%	53 (18%)	14%, 23%	-37%	-51%, -23%	< 0.001	< 0.001
Provider conducted cervical lymph node examination	18 (27%)	17%, 39%	38 (13%)	9.5%, 18%	-14%	-26%, -1.6%	0.008	0.7
Provider conducted chest examination with a stethoscope	60 (90%)	79%, 95%	248 (85%)	80%, 89%	-4.6%	-14%, 4.7%	0.4	> 0.9
Provider measured body weight	17 (25%)	16%, 38%	90 (31%)	26%, 37%	5.4%	-7.2%, 18%	0.5	> 0.9
Provider measured body height	0 (0%)	0.00%, 6.8%	7 (2.4%)	1.1%, 5.1%	2.4%	-0.27%, 5.1%	0.4	> 0.9
**Tests and Examinations Recommended**								
Any diagnostic test	48 (72%)	59%, 82%	234 (80%)	75%, 84%	8.5%	-4.1%, 21%	0.2	> 0.9
Any TB test (Chest X-ray/Sputum test)	48 (72%)	59%, 82%	216 (74%)	68%, 79%	2.3%	-10%, 15%	0.8	> 0.9
Any TB sputum test	19 (28%)	18%, 41%	86 (29%)	24%, 35%	1.1%	-12%, 14%	> 0.9	> 0.9
Chest x-ray	44 (66%)	53%, 77%	204 (70%)	64%, 75%	4.2%	-9.3%, 18%	0.6	> 0.9
Xpert	0 (0%)	0.00%, 6.8%	3 (1.0%)	0.27%, 3.2%	1.0%	-1.0%, 3.1%	> 0.9	> 0.9
Sputum microscopic examination/Acid Fast Bacilli test	19 (28%)	18%, 41%	81 (28%)	23%, 33%	-0.62%	-13%, 12%	> 0.9	> 0.9
Sputum culture	0 (0%)	0.00%, 6.8%	4 (1.4%)	0.44%, 3.7%	1.4%	-0.88%, 3.6%	0.8	> 0.9
Drug Sensitivity Testing	0 (0%)	0.00%, 6.8%	1 (0.3%)	0.02%, 2.2%	0.34%	-0.67%, 1.4%	> 0.9	> 0.9
Routine hematology test	7 (10%)	4.7%, 21%	44 (15%)	11%, 20%	4.6%	-4.7%, 14%	0.4	> 0.9
Erythrocyte Sedimentation Rate	3 (4.5%)	1.2%, 13%	17 (5.8%)	3.5%, 9.3%	1.3%	-5.2%, 7.9%	0.9	> 0.9
TB Serology test (IGRA, etc.)	0 (0%)	0.00%, 6.8%	1 (0.3%)	0.02%, 2.2%	0.34%	-0.67%, 1.4%	> 0.9	> 0.9
Diabetes test/glucose test	2 (3.0%)	0.52%, 11%	4 (1.4%)	0.44%, 3.7%	-1.6%	-6.8%, 3.6%	0.7	> 0.9
Other test	4 (6.0%)	1.9%, 15%	9 (3.1%)	1.5%, 6.0%	-2.9%	-9.8%, 4.0%	0.4	> 0.9
Type of facility recommended for TB test referral								
This HCF	16 (5.5%)	3.3%, 8.9%	5 (7.5%)	2.8%, 17%				
Private HCF not linked to NTP	64 (22%)	17%, 27%	27 (40%)	29%, 53%				
Public HCF linked to NTP	27 (9.2%)	6.3%, 13%	6 (9.0%)	3.7%, 19%				
Public HCF not linked to NTP	8 (2.7%)	1.3%, 5.5%	0 (0%)	0.00%, 6.8%				
Private HCF linked to NTP	2 (0.7%)	0.12%, 2.7%	1 (1.5%)	0.08%, 9.1%				
More than one HCF type	32 (11%)	7.7%, 15%	5 (7.5%)	2.8%, 17%				
Not specified	67 (23%)	18%, 28%	4 (6.0%)	1.9%, 15%				
Not applicable	76 (26%)	21%, 32%	19 (28%)	18%, 41%				
Type of facility recommended for all test referrals								
This HCF	20 (6.8%)	4.3%, 11%	5 (7.5%)	2.8%, 17%				
Private HCF not linked to NTP	56 (19%)	15%, 24%	26 (39%)	27%, 52%				
Public HCF linked to NTP	29 (9.9%)	6.9%, 14%	6 (9.0%)	3.7%, 19%				
Public HCF not linked to NTP	7 (2.4%)	1.1%, 5.1%	0 (0%)	0.00%, 6.8%				
Private HCF linked to NTP	2 (0.7%)	0.12%, 2.7%	1 (1.5%)	0.08%, 9.1%				
More than one HCF type	49 (17%)	13%, 22%	6 (9.0%)	3.7%, 19%				
Not specified	71 (24%)	20%, 30%	4 (6.0%)	1.9%, 15%				
Not applicable	58 (20%)	16%, 25%	19 (28%)	18%, 41%				
**Diagnosis and Counseling**								
Working diagnosis given by the provider								
Tuberculosis	40 (60%)	47%, 71%	133 (46%)	40%, 51%				
Upper respiratory infection	6 (9.0%)	3.7%, 19%	52 (18%)	14%, 23%				
No diagnosis given	16 (24%)	15%, 36%	37 (13%)	9.2%, 17%				
Lower respiratory infection, unspecified	1 (1.5%)	0.08%, 9.1%	37 (13%)	9.2%, 17%				
Lower respiratory infection, bronchitis	2 (3.0%)	0.52%, 11%	14 (4.8%)	2.7%, 8.1%				
Allergy	2 (3.0%)	0.52%, 11%	10 (3.4%)	1.7%, 6.4%				
Lower respiratory infection, COVID-19	NA	NA	7 (2.4%)	1.1%, 5.1%				
Heart disease	0 (0%)	0.00%, 6.8%	1 (0.3%)	0.02%, 2.2%				
Occupational fatigue	0 (0%)	0.00%, 6.8%	1 (0.3%)	0.02%, 2.2%				
Provider asked SP to return…	51 (76%)	64%, 85%	164 (56%)	50%, 62%	-20%	-33%, -7.3%	0.004	0.4
…if symptoms persist or worsen	22 (43%)	30%, 58%	83 (51%)	43%, 58%	7.5%	-9.4%, 24%	0.4	> 0.9
…to take medication	7 (14%)	6.2%, 27%	6 (3.7%)	1.5%, 8.2%	-10%	-21%, 1.1%	0.022	> 0.9
…to receive lab test results	41 (80%)	66%, 90%	99 (60%)	52%, 68%	-20%	-35%, -5.5%	0.014	> 0.9
…for another reason	3 (5.9%)	1.5%, 17%	26 (16%)	11%, 23%	10.0%	0.15%, 20%	0.11	> 0.9
Provider explained the duration of treatment given	36 (54%)	41%, 66%	147 (50%)	44%, 56%	-3.4%	-18%, 11%	0.7	> 0.9
Provider explained the importance of taking medicine regularly	28 (42%)	30%, 54%	101 (35%)	29%, 40%	-7.2%	-21%, 6.7%	0.3	> 0.9
Provider explained the importance of undergoing treatment to completion	22 (33%)	22%, 46%	99 (34%)	29%, 40%	1.1%	-12%, 14%	> 0.9	> 0.9
Provider explained the side effects that could arise from the prescribed medication	5 (7.5%)	2.8%, 17%	15 (5.1%)	3.0%, 8.5%	-2.3%	-10%, 5.4%	0.7	> 0.9
Provider explained cough etiquette	5 (7.5%)	2.8%, 17%	13 (4.5%)	2.5%, 7.7%	-3.0%	-11%, 4.6%	0.5	> 0.9
Provider explained the importance of quitting smoking	16 (24%)	15%, 36%	52 (18%)	14%, 23%	-6.1%	-18%, 6.0%	0.3	> 0.9
Provider advised SP to register for Indonesia national health insurance	9 (13%)	6.7%, 24%	17 (5.8%)	3.5%, 9.3%	-7.6%	-17%, 1.9%	0.057	> 0.9
**Prescriptions**								
Provider prescribed and/or dispensed medication	60 (90%)	79%, 95%	279 (96%)	92%, 98%	6.0%	-2.6%, 15%	0.10	> 0.9
Number of medications prescribed	3.23 (1.01)	3.0, 3.5	3.38 (0.96)	3.3, 3.5	0.15	-0.14, 0.43	0.3	> 0.9
Provider prescribed non- ATT antibiotics	49 (73%)	61%, 83%	198 (68%)	62%, 73%	-5.3%	-18%, 7.5%	0.5	> 0.9
Cephalosporin and other beta lactam antibiotics	17 (25%)	16%, 38%	121 (41%)	36%, 47%	16%	3.3%, 29%	0.022	> 0.9
Penicillin	12 (18%)	10.0%, 30%	17 (5.8%)	3.5%, 9.3%	-12%	-23%, -1.6%	0.002	0.2
Quinolones: ciprofloxacin, ofloxacin	8 (12%)	5.7%, 23%	27 (9.2%)	6.3%, 13%	-2.7%	-12%, 6.7%	0.7	> 0.9
Macrolide	8 (12%)	5.7%, 23%	26 (8.9%)	6.0%, 13%	-3.0%	-12%, 6.3%	0.6	> 0.9
Sulfonamide and trimethoprim	1 (1.5%)	0.08%, 9.1%	3 (1.0%)	0.27%, 3.2%	-0.47%	-4.1%, 3.1%	> 0.9	> 0.9
Tetracycline	0 (0%)	0.00%, 6.8%	1 (0.3%)	0.02%, 2.2%	0.34%	-0.67%, 1.4%	> 0.9	> 0.9
Other antibiotics	3 (4.5%)	1.2%, 13%	4 (1.4%)	0.44%, 3.7%	-3.1%	-9.2%, 2.9%	0.2	> 0.9
Provider prescribed First-Line ATT antibiotics (Rifampicin, Isoniazid, Ethambutol, or Pyrazinamide)	2 (3.0%)	0.52%, 11%	0 (0%)	0.00%, 1.6%	-3.0%	-8.0%, 2.0%	0.040	> 0.9
Provider prescribed Second Line ATT antibiotics (Levofloxacin)	3 (4.5%)	1.2%, 13%	31 (11%)	7.4%, 15%	6.1%	-0.86%, 13%	0.2	> 0.9
Provider prescribed anti-inflammation medication (Corticosteroids)	21 (31%)	21%, 44%	78 (27%)	22%, 32%	-4.6%	-18%, 8.5%	0.5	> 0.9
Provider prescribed vitamin	7 (10%)	4.7%, 21%	110 (38%)	32%, 44%	27%	17%, 37%	< 0.001	0.003
Provider prescribed symptomatic/over the counter medication	59 (88%)	77%, 94%	207 (71%)	65%, 76%	-17%	-27%, -6.9%	0.006	0.5
Provider dispensed drug with no label	5 (7.5%)	2.8%, 17%	48 (16%)	12%, 21%	9.0%	0.46%, 17%	0.094	> 0.9
**Costs**								
Total cost of entire visit (IDR)	119,413 (84,544)	98,791, 140,035	121,869 (75,417)	113,182, 130,555	2,456	-19,853, 24,765	0.8	> 0.9
Total cost of entire visit (USD)	8.30 (5.90)	6.90, 9.80	8.40 (5.20)	7.80, 9.00	0.13	-1.40, 1.70	0.9	> 0.9

Acronyms: *INSTEP* Investigation of services delivered for TB by external care system - especially the private sector, *COVET* COVID Effects on TB Services in the Private Sector, *TB* Tuberculosis, *SP* Standardized Patient, *NTP* Indonesian National Tuberculosis Programme, *HIV* Human immunodeficiency virus, *IGRA* Interferon-Gamma Release Assay, *COVID-19* Coronavirus disease 2019, *ATT* Anti-tuberculosis treatment, *IDR* Indonesian Rupiah, *USD* United States Dollar

^a^Mean (SD); n (%)

^b^*CI* Confidence Interval

^c^Welch Two Sample t-test; Two sample test for equality of proportions

^d^Bonferroni-Holm correction for multiple testing

#### Provider tests and referrals

Providers recommended a diagnostic test in 80% of visits. The most recommended tests were chest X-ray (70%) and any sputum test (29%). Xpert was recommended in three SP visits (1%). Providers referred SPs to other facilities in 21% of visits. The most common place for referral was a public DOTS center (72% of referrals, 44/61).

A working diagnosis was communicated to SPs in 87% of visits. More than half of diagnoses mentioned to SPs were for TB (52% of diagnosed visits, 133/255). Other diagnoses mentioned included upper respiratory infection (20%, 52/255), unspecified lower respiratory infection (15%, 37/255), and bronchitis (5.5%, 14/255).

#### Prescriptions

The provider prescribed or dispensed medication in 96% of visits. On average, each SP was prescribed three medications (SD: 1.17). The most prescribed drugs were over the counter symptomatic drugs (71%) and non-anti-tuberculosis treatment (ATT) antibiotics (68%). Corticosteroids were prescribed in 27% of visits. Unlabeled drugs were dispensed in 16% of visits. While no providers prescribed first-line ATT drugs, 11% of providers prescribed levofloxacin, a second-line ATT drug.

#### COVID-19 questions

Providers asked about anosmia (impaired smell) in 28% of visits and ageusia (impaired taste) in 15% of visits. Providers told the SP they might have COVID-19 in 14% of visits and named COVID-19 as a likely diagnosis in 8% of visits. A COVID-19 test (rapid antibody, antigen, or polymerase chain reaction (PCR) test) was recommended in 20% of visits. Detailed information about the SP visits related to COVID-19 can be found in Supplementary Table S3.

### Are private providers in Bandung correctly managing people with TB symptoms?

Overall, 35% of interactions were managed in accordance with the 2016 Indonesia NTP guidelines (95% CI: 29.2–40.4%). However, 81% of interactions were managed correctly according to the definition of correct management used in prior SP studies (95% CI: 75.7–85.1%).

### Which types of private providers are more likely to correctly manage people with TB symptoms?

Table 4 shows the adjusted odds ratios (aORs) representing the association between selected provider, facility, and visit characteristics and correct management according to the Indonesian NTP guidelines (defined as bacteriological examination by sputum smear or Xpert, or referral to DOTS center). The likelihood of appropriate TB management decreased with each 5-year increase in provider age (aOR: 0.76, 95% CI: 0.67–0.87, *p* < 0.001) and among male providers, although the latter is not statistically significant (aOR: 0.52, 95% CI: 0.27–1.01, *p* = 0.052). Providers who asked more questions in the history-taking portion of the visit were more likely to manage the case according to NTP guidelines (aOR: 1.07, 95% CI: 1.02–1.13, *p* = 0.004) as were those who asked about cardinal TB symptoms during the visit (aOR: 2.78, 95% CI: 1.82–4.23, *p* < 0.001). Providers who prescribed broad-spectrum or non-ATT antibiotics were negatively associated with appropriate TB management, though this association was not statistically significant (aOR: 0.56, 95% CI: 0.32–1.00, *p* = 0.051).

**Table 4:** Factors associated with correct management (NTP 2016 guidelines) of presumptive TB case in COVET study

Characteristic	Adjusted odds ratio (aOR)^a^	95% Confidence Interval (CI)^a^	*P *value
**Provider characteristics**			
Provider age increase of 5 years^b^	0.76	0.67, 0.87	< 0.001
Provider sex = Male^c^ (reference = Female)	0.52	0.27, 1.01	0.052
Provider qualification = Specialist^d ^(reference = General Practitioner)	0.84	0.22, 3.21	0.8
Provider received training on TB management^e ^(reference = no training)	0.95	0.53, 1.71	0.9
Provider diagnoses at least one TB case per month^f ^(reference = no diagnoses)	0.88	0.50, 1.56	0.7
**Facility characteristics**			
Facility type = Solo Practice^g ^(reference = Clinic/Hospital)	0.78	0.32, 1.87	0.6
Sputum examination available at facility^h ^(reference = sputum examination not available)	0.56	0.17, 1.84	0.3
**Visit characteristics**			
Visit length increase of 5 min^i^	1.05	0.84, 1.31	0.7
Visit cost increase of $5 (USD)^h^	0.81	0.59, 1.10	0.2
Number of questions asked in history-taking^e^ (continuous)	1.07	1.02, 1.13	0.004
Number of cardinal TB symptoms asked in visit^e^ (continuous)	2.78	1.82, 4.23	< 0.001
Provider prescribed non-ATT antibiotic^e ^(reference = did not prescribe non-ATT antibiotic)	0.56	0.32, 1.00	0.051
Provider prescribed steroids^e ^(reference = did not prescribe steroids)	0.74	0.40, 1.37	0.4

Acronyms: *TB* Tuberculosis, *USD* United States Dollar, *ATT* Anti-tuberculosis treatment

^a^*aOR* Adjusted Odds Ratio, *CI* Confidence Interval

^b^Adjusted by provider sex

^c^Adjusted by SP sex

^d^Adjusted by provider age, provider sex, and facility type

^e^Adjusted by provider age and qualification

^f^Adjusted by provider age, qualification, and provider received training on TB management

^g^Adjusted by provider age

^h^Adjusted by provider qualification and facility type

^i^Adjusted by number of questions asked in history-taking

### What is the extent of changes in clinical practices in TB care during the COVID-19 pandemic?

Sixty-seven SP visits made to private providers in Bandung between 6 July 2018 and 1 April 2019 were compared to the 292 SP visits made to private providers between 9 July 2021 and 21 January 2022 in the same sampling area and for the same SP case. Table 3 compares provider, facility, and SP visit characteristics between these two studies. Table 5 lists adjusted odds ratios comparing proportions of main outcomes across the two studies.

**Table 5:** Regressions on outcomes of interest comparing INSTEP and COVET studies

**Binary Outcomes**	**N**^**a**^	**aOR**^**b**^	**95% CI**^**c**^	***P*-value**^**d**^
NTP 2016 Guidelines	340	0.97	0.44, 2.14	> 0.90
Correct management based on prior SP studies	340	0.74	0.00, 113	> 0.90
Recommendation for any TB test	340	1.09	0.53, 2.24	> 0.90
Recommendation for chest X-ray	340	1.35	0.67, 2.71	> 0.90
Recommendation for any sputum test	340	0.73	0.33, 1.60	> 0.90
Prescribed steroids	340	0.74	0.34, 1.58	> 0.90
Prescribed other non-ATT antibiotics	340	0.70	0.35, 1.42	> 0.90
Checked SP’s temperature	340	8.05	2.96, 21.9	< 0.001
Conducted throat examination	340	0.16	0.06, 0.41	0.002
Examined SP’s lymph nodes	340	0.41	0.18, 0.94	0.43
Referral to public DOTS	340	0.70	0.27, 1.83	> 0.90
Request to come back	340	0.43	0.20, 0.94	0.43
**Linear Outcomes**	**N**^**a**^	**Beta**	**95% CI**^**c**^	***P*-value**^**d**^
Length of visit (minutes)	340	-0.32	-2.5, 1.9	> 0.90
Number of questions asked	340	-0.91	-2.2, 0.37	> 0.90
Number of exams	340	0.01	-0.52, 0.54	> 0.90
Cost of visit (USD)^e^	340	1.6	0.39, 2.9	0.15

Acronyms: *INSTEP* Investigation of services delivered for TB by external care system - especially the private sector, *COVET* COVID Effects on TB Services in the Private Sector, *NTP* National Tuberculosis Programme, *SP* Standardized Patient, *TB* Tuberculosis, *ATT* Anti-tuberculosis treatment, *DOTS* Directly Observed Treatment Shortcourse, *USD* United States Dollar

^a^19 observations removed due to missing age (13 in INSTEP and 6 in COVET)

^b^*aOR* Adjusted Odds Ratio of outcomes described by study year, controlled by provider qualification and age (reference = 2018 INSTEP study)

^c^*CI* Confidence Interval

^d^Adjusted using the Bonferroni-Holm method

^e^Additionally controlled by whether the provider prescribed medication

We did not observe significant differences in the proportion of SP visits managed in concordance to the 2016 NTP guidelines (difference between INSTEP and COVET: 6.2%, 95% CI: -6.8–19%) nor correct management based on prior SP studies (difference: 9.2%, 95% CI: -3.4–22%). More providers measured the SP’s temperature in the study conducted during COVID-19 (aOR 8.05, 95% CI: 2.96–21.9, *p* < 0.001). Fewer providers conducted throat examinations in the during COVID-19 study (aOR 0.16, 95% CI: 0.06–0.41, *p* = 0.002).

## Discussion

This SP study is one of the first that examines TB management before and after the start of the COVID-19 pandemic in the same geographical area. We draw four main conclusions from the results.

Our first main finding is that the management of presumptive TB cases by private providers in Bandung, Indonesia, is comparable to (or even better than) previous SP study findings from other countries. Specifically, adherence to expected TB management based on prior SP studies in this sample (81%) was notably higher than SP studies of private practitioners conducted in Kenya (33%), India (35% and 16%), South Africa (43%), and Nigeria (56%) [20–23]. The fact that 70% of SPs in this sample were recommended for a chest X-ray and that private providers named TB as the working diagnosis in 46% of all visits are indications that most private providers in this setting are identifying that their patients have a lung infection that could be TB. Nevertheless, we also observed a high usage of unnecessary antibiotics, similar to SP studies in South Africa, India, and China [22, 34, 35], as well as much higher steroid use in this sample (27%) compared to private practitioners in India (2%), Nigeria (3%), China (5%), and South Africa (7.1%) [22, 23, 34, 36]. Private providers thus seem to know how to identify presumptive TB, but they are not following the NTP guidelines and tend to overuse antibiotics and steroids.

Our second main finding is that the proportion correctly managed according to NTP guidelines remains low at 32% for private GPs and 26% for private specialists. The 2016 NTP guidelines require microbiological confirmation via Xpert testing and/or sputum tests rather than the use of chest X-ray [33]. Xpert testing among private providers remains very low at 1% of all visits. One reason could be that implementation of the 2021 NTP guidelines has slowed due to the COVID-19 pandemic-related resources diversion and the limited availability of Xpert machines in Bandung. At present, Bandung has 16 Xpert machines located only at Puskesmas and public hospitals (source: Bandung District Health Office, unpublished data). Sputum microscopy by private providers is also rare at 29% (30% of GPs, 19% of specialists). These results contradict findings from studies that use telephone surveys of Indonesian private providers to estimate their rate of referral for various methods of TB diagnosis, one of which found that 74.1% of private providers in Bandung utilized smear microscopy in diagnosing TB [14]. SP studies from other contexts including India, South Africa, and China have found similar results of low smear microscopy utilization [20, 22, 34, 36]. Stronger private provider engagement on the importance of Xpert and sputum microbiological testing over chest X-ray could result in private providers diagnosing more TB cases. Additional efforts may need to be considered such as easier and free access to public-sector Xpert testing for patients managed in the private sector.

Our third main finding is that we observed positive associations between appropriate TB management and asking more questions in the history-taking portion of the visit, especially inquiring about cardinal TB symptoms, and negative associations between appropriate management and increasing provider age. These findings are in line with those from other SP studies conducted in South Africa [22] and China [36], respectively. Asking more and specific questions about TB symptoms may be an indication that the provider suspects TB. Furthermore, providers who know the signs and symptoms of TB may also know how to appropriately manage TB cases. Interestingly, we did not see a significant association between our appropriate management and visit length, which we might expect to see if providers who ask more history-taking questions may take more time in their consultations compared to providers who ask fewer questions; this was also the case in the SP study conducted in South Africa [22]. The negative correlation between age and correct management may be an indicator of improved education for younger providers on the importance of referral for appropriate testing of people with common TB symptoms. We did not, however, observe an association between previous provider training in TB and correct management. As this indicator was self-reported by providers in a survey conducted during the mapping exercise that preceded SP visits, associations between our outcome and previous provider training in TB could be masked by information biases. Further studies may need to be conducted to appropriately estimate the importance of formal education and on-the-job training on correct management of presumptive TB cases.

Our fourth main finding is that the quality of TB care among private practitioners, as defined by Indonesian national guidelines, has not dropped in the COVID-19 era. This may provide some relief to the concern that providers would mistake TB cases for COVID-19 cases given the overlap in symptoms. While our findings provide some evidence to support this concern, as 14% of providers indeed suspected that the SPs had COVID-19, 65% (26/40) of these providers also recommended a chest X-ray or related TB test. This may indicate that providers are still considering TB even when they suspect that the SP has COVID-19. Furthermore, we find limited evidence to support that private hospitals and clinics are engaged in bi-directional screening of TB and COVID-19, despite strong government support [9]. Providers asked at least one COVID-19 related questions in less than 20% of cases. This suggests that only some private providers have changed their practices to incorporate COVID-19 as a differential diagnosis for this case presentation.

The fact that providers suspect that they are dealing with a patient who might have TB but are not following the correct NTP guidelines suggests that private providers may not be properly exposed to the NTP guidelines nor sensitized on the importance of following these guidelines. Private provider engagement has been limited in Indonesia to date and has focused primarily on private hospital linkage to DOTS [37, 38]. In 2021, the Indonesia Medical Association announced they would begin offering continuing education credits as a reward to providers who notify TB patients to the NTP [39]. Proper engagement of private providers through initiatives like these could result in increased TB diagnoses and notifications by the private health sector, as has been seen elsewhere in Indonesia [40] and in other similar settings [41–43]. Expansion of diagnostic tools into private facilities or implementation of expedited referral systems could also accelerate improvement in this area, as previous studies have indicated that patients often refuse referrals to the public sector due to lack of convenience, long waiting times, and a lack of trust in the public system [40]. It has been well-established that people with TB in Indonesia prefer to visit private providers, despite these services costing more than the public sector [44, 45]. Improving the quality of care among private providers and the connection of private providers to the NTP is a practical and person-centred approach, responding to patients’ needs and preferences.

Our study has several limitations. First, SPs are simulations of real patients, not actual patients. The standardization of the case is what allows for valid inference, but with real patients we would likely see far more variation in how patients and physicians behave. The SP methodology for presumptive TB also does not allow for repeated visits, so we have not observed how providers would behave if the SP were to return for a second visit. This may not be as severe a limitation as typically believed as a recent SP study has established the validity of the single-visit protocol in this case [46]. Second, only providers who consented to the provider mapping survey were included in the SP study (14% refusal rate, Fig. 1). This is a potential source of selection bias. Furthermore, only the presumptive TB case scenario was used in this study and could only be compared to a small subset of SP visits conducted in the INSTEP SP study, which limited the statistical power to detect major differences between these two samples. Third, we present here results of a comparison of two cross-sectional studies, but the study is not designed to estimate the causal impact of COVID-19 on provider behavior as different from other general time trends, or parallel program implementation efforts. We were also limited in terms of our sample size, which only allowed detection of a 15% or greater change in TB management between the two studies. Finally, the results presented here only measure provider behavior in a finite time during the COVID-19 pandemic. Due to the variable nature of the pandemic, these results cannot be generalized to the entire COVID-19 pandemic.

## Conclusions

Findings from this study reveal severe gaps in management of presumptive TB cases by private providers in Bandung, Indonesia. Results from this study indicate that private providers successfully identify TB in their patients yet do not manage them properly. There is great potential yet to be tapped in the Indonesian private health sector to find the missing TB cases and reduce delays in diagnosing people with TB.

### Supplementary Information

**Additional file 1: Supplement 1.** Standardized patient case scenario and script. **Supplement 2.** Details on confounder selection. **Supplementary Table S3.** Characteristics of the SP visits related to COVID-19 (COVET study only).

## Data Availability

The datasets used during the current study are available from the corresponding author on reasonable request.

## Abbreviations

AFB: Acid–fast bacilli
aOR: Adjusted odds ratio
ATT: Anti–tuberculosis treatment
CHC: Community health center
CI: Confidence interval
COVET: COVID Effects on TB Services in the Private Sector
COVID-19: Coronavirus disease of 2019
DAG: Directed acyclic graph
DOTS: Directly observed therapy, shortcourse
DST: Drug susceptibility testing
HIV: Human immunodeficiency virus
IDR: Indonesian rupiah
INSTEP: Investigation of services delivered for TB by external care system–especially the private sector
ISTC: International Standard of Tuberculosis Care
JKN: *Jaminan Kesehatan Nasional*
MTB: Mycobacterium tuberculosis
MUHC: McGill University Health Centre
NTP: National Tuberculosis Programme
PCR: Polymerase chain reaction
REB: Research Ethics Board
REDCap: Research Electronic Data Capture
SD: Standard deviation
SP: Standardized patient
TB: Tuberculosis
USD: United States dollar
Xpert: GeneXpert MTB/Rif
