# Supplementary material for: Impact of the COVID-19 pandemic on quality of tuberculosis care in private facilities in Bandung, Indonesia: a repeated cross-sectional standardized patients study
Source: BMC Public Health. 2024 Jan 5;24:102. doi: 10.1186/s12889-023-17001-y (PMC10771004; doi:10.1186/s12889-023-17001-y)
Supplement: Supplementary file 1 — Additional file 1: Supplement 1. Standardized patient case scenario and script. Supplement 2. Details on confounder selection. Supplementary Table S3. Characteristics of the SP visits related to COVID-19 (COVET study only). [file 12889_2023_17001_MOESM1_ESM.docx]

# Table of Contents

[Supplement 1: Standardized patient case scenario and script 2](#_Toc138932739)

[Supplement 2: Details on confounder selection 6](#_Toc138932740)

[Supplement 3: Characteristics of the SP visits related to COVID-19 (COVET study only) 16](#_Toc138932741)

# Supplement 1: Standardized patient case scenario and script

**NARRATIVE CASE 1 (SUSPECTIVE TB)**

**Chief Complaint: Cough**

A classic case of suspected tuberculosis with a cough lasting 2-3 weeks, low-grade fever, cold sweats, loss of appetite and other typical symptoms of TB. The patient has no history of COVID-19 and has never been tested for COVID-19. The patient has received one dose of a COVID-19 vaccine.

**CASE SCENARIO 1:**

X (name according to Kartu Tanda Penduduk [KTP, Indonesia ID card]), a male/female (age according to KTP), is a high school graduate who works as an employee in a plastic shop (customized) at the kosambi market in Bandung. Currently X lives in a boarding house in the Ahmad Yani area (adjusted to the area of the visit). X earns a monthly salary of IDR 2,150,000 from the shop where he/she works. X does not carry an ID card/other identity card and does not have health insurance (BPJS or private insurance).

Since the last 2-3 weeks, X has had a persistent cough. Cough with yellowish phlegm. Sputum is not accompanied by blood. X also complains of body heat that is not too high, intermittent and irregular. X feels sweaty at night so he/she has to get up and change clothes. In addition, there are complaints such as fatigue, decreased appetite and weight loss so that X's clothes feel looser.

X does not experience sore throat, runny nose/stuffy nose, wheezing when breathing, shortness of breath, chest pain, headache, muscle aches, nausea, vomiting, diarrhea, or decreased ability to smell and taste.

X has treated his/her complaint by taking OBH (*Obat Batuk Hitam*, cough syrup) cough medicine plus panadol (brand name of paracetamol/acetaminophen) for 1 week but the cough has not reduced. After taking Panadol the fever disappeared and then reappeared. X does not take antibiotics.

Previously, X had no health problems or other chronic diseases, nor did his/her family. X started smoking when he/she was about 18 years old. In one day, X can smoke up to half a pack. Since the onset of cough 2-3 weeks, X began to reduce smoking habits, from half a pack to 2-3 cigarettes per day, sometimes not smoking for a whole day. However, the complaint of cough was still felt by X.

At work, since the pandemic, X always wears a mask and keeps a distance from other employees. The shop's work schedule is always divided into 2 morning and evening shifts (one day at most 2 employees, usually 4 employees). X has never been in contact with a confirmed case of covid. There is no history of traveling in the last 2 weeks outside the city or COVID red zone area. X does not feel sore throat, flu, muscle aches, reduced smell or taste. X has never done a swab test, neither antigen nor PCR.

**No one has similar complaints in the family or at work**. However, X's roommate is known to have frequent coughs and has been receiving treatment for pulmonary TB at the Puskesmas for the last 3 months.

X has good relationships with his/her family and friends. Previously, X was a man/woman with a pleasant personality, but today, X's face looks tense due to worry about the cough and fever he/she is experiencing, which makes him/her go to an independent practice doctor.

The following dialog is based on case scenario 1:

Opening: The standardized patient (SP) complains: "Doctor, I have a cough."

| **Question Number** | **Speaker** | **Line** |
| --- | --- | --- |
| **MAIN COMPLAINT** | | |
| P1 | Doctor | How long has the cough been? |
|  | Standardized Patient | It's been about 3 weeks, Doc. |
| P2 | Doctor | Does the cough have phlegm, sir? |
|  | Standardized Patient | There is. |
| P3 | Doctor | What color is the phlegm? |
|  | Standardized Patient | The color is a bit yellow, Doc. |
| P4 | Doctor | Are there blood clots/spots in the sputum? |
|  | Standardized Patient | There isn't any. |
| P5 | Doctor | Does the cough feel continuous/all day? |
|  | Standardized Patient | Yes, Doc. |
|  | P6 |  |
| P6 | Doctor | Any fever? |
|  | Standardized Patient | There is, Doc |
| P7 | Doctor | How long has the fever been? |
|  | Standardized Patient | It's been 2-3 weeks, Doc. |
| P8 | Doctor | How's the fever? With chills, high, just warm? |
|  | Standardized Patient | Just warm, Doc |
| P9 | Doctor | Did the fever come on suddenly or does it get high? |
|  | Standardized Patient | Not sure, Doc |
| P10 | Doctor | Is the fever constant (all day) or does it come and go? |
|  | Standardized Patient | Coming and going, Doc, before sunset to night. |
| P11 | Doctor | Do you have night sweats even when you're not active? |
|  | Standardized Patient | Yes, Doc, sometimes I have to change clothes. |
| P12 | Doctor | How's your appetite now? (Appetite reduced?) |
|  | Standardized Patient | Less appetite now, Doc. (Yes Doc) |
| P13 | Doctor | Have you lost weight recently? |
|  | Standardized Patient | I don't know Doc, I've never weighed it, but I think so, Doc, because lately my pants have been feeling looser. |
| P14 | Doctor | Do you feel weaker/tired easily than usual? |
|  | Standardized Patient | Yes, Doc, it's easier to get tired. |
| **HISTORY OF CURRENT DISEASE** | | |
| P15 | Doctor | Do you have a sore throat? |
|  | Standardized Patient | No, Doc. |
| P16 | Doctor | Do you have a runny nose/stuffy nose? |
|  | Standardized Patient | No, Doc |
| P17 | Doctor | Do you have wheezing / breath sounds / wheezy? |
|  | Standardized Patient | No, there isn't any. |
| P18 | Doctor | Do you have shortness of breath? |
|  | Standardized Patient | No shortness of breath |
| P19 | Doctor | Do you have chest pain? |
|  | Standardized Patient | No, Doc. |
| P20 | Doctor | Are you having a headache? |
|  | Standardized Patient | No, Doc. |
| P21 | Doctor | Do you have muscle pain? |
|  | Standardized Patient | No, Doc |
| P22 | Doctor | Are you experiencing nausea and vomiting? |
|  | Standardized Patient | No, Doc |
| P23 | Doctor | Do you have digestive disorders such as diarrhea? |
|  | Standardized Patient | No, Doc. |
| P24 | Doctor | Are you experiencing any loss of smell? |
|  | Standardized Patient | No, Doc. |
| P25 | Doctor | Are you experiencing any loss of taste? |
|  | Standardized Patient | No, Doc. |
| **TREATMENT HISTORY** | | |
| P26 | Doctor | Did you take medicine before coming here? |
|  | Standardized Patient | Yes, Doc |
| P27 | Doctor | What's the name of the medicine? |
|  | Standardized Patient | I bought OBH with panadol. |
| P28 | Doctor | How long have you been taking the medicine? |
|  | Standardized Patient | One week, Doc. |
| P29 | Doctor | Are you taking antibiotics? |
|  | Standardized Patient | No, Doc. |
| P30 | Doctor | Are you taking certain drugs for a long time for other diseases or not? |
|  | Standardized Patient | No, Doc. |
| **HISTORY OF EXPOSURE TO COVID** | | |
| P31 | Doctor | Have you ever been to an area with high COVID cases/Red Zone? |
|  | Standardized Patient | Never, Doc. |
| P32 | Doctor | Have you been in close contact with a person who is positive for COVID? (eating together, staying overnight, gathering in a closed room for more than 15 minutes without a mask) |
|  | Standardized Patient | Never, Doc. |
| P33 | Doctor | Have you ever had a swab test? |
|  | Standardized Patient | Not yet, Doc. |
| P34 | Doctor | Have you ever had COVID? |
|  | Standardized Patient | Never, Doc. |
| **HISTORY OF EXPOSURE TO TB** | | |
| P35 | Doctor | Is there a family member who has lung disease/TB/lung spots/wet lungs (experiencing the same complaint as you)? |
|  | Standardized Patient | Nothing, Doc. But a friend from my boarding house has a lung disease. Now being treated at the Puskesmas, it has been three months. |
| P36 | Doctor | Have any family members and/or close people ever suffered from TB/spots/wet lung disease? |
|  | Standardized Patient | No, Doc. |
| P37 | Doctor | Who do you live at home with? There are no children? |
|  | Standardized Patient | I live in a boarding house, if I have a toddler at home, it’s my nephew visiting. |
| **HISTORY OF PREVIOUS DISEASES** | | |
| P38 | Doctor | Have you ever had TB/lung spots/wet lungs before? |
|  | Standardized Patient | Never. |
| P39 | Doctor | Have you ever been treated for TB/lung spots/wet lungs before (drugs that have to be taken for 6-9 months and make urine red)? |
|  | Standardized Patient | Never. |
| P40 | Doctor | Do you have any drug allergies? If you take medicine, you will get itchy red |
|  | Standardized Patient | Never, Doc. |
| P41 | Doctor | Do you have diabetes? |
|  | Standardized Patient | Don't know. |
| P42 | Doctor | Have you ever been told by a doctor that you have HIV/AIDS? |
|  | Standardized Patient | Don't know. |
| P43 | Doctor | Do you have hypertension/high blood pressure? |
|  | Standardized Patient | Don't know. |
| P44 | Doctor | Do you suffer from other illnesses besides the current complaint? |
|  | Standardized Patient | No Doc, just healthy before. |
| P45 | Doctor | Do you smoke? |
|  | Standardized Patient | Yes, I smoke. |
| P46 | Doctor | How much do you smoke in one day? |
|  | Standardized Patient | Usually half a pack a day, but since this illness I have reduced it to 2-3 cigarettes per day. |
| P47 | Doctor | Do you have a habit of consuming alcoholic beverages? |
|  | Standardized Patient | No, Doc. |
| P48 | Standardized Patient | Sorry, doc, let me ask, so what do you think I'm sick of? |
|  | Doctor | For a while I can't be sure, I'm suspicious towards TB……. |

# Supplement 2: Details on confounder selection

Figure S2-a depicts the directed acyclic graph (DAG) showing relationships between relevant variables in the SP visits conducted in this study, made using DAGitty, a browser-based environment for creating, editing, and analyzing causal diagrams. DAGs are best practice among studies attempting to quantify causal relationships between exposures and outcomes (1). DAGs are not typically used for confounder selection in cross-sectional studies such as this one, however, we were interested in several “exposure”-outcome relationships in our sample pertaining to our second research question (Which types of private providers are more likely to correctly manage people with TB symptoms?). Given the complex nature of the relationships among these variables, we asserted that the best way to assess these given “exposures” would be through regression models for each exposure which contain appropriate confounders chosen from a DAG, in particular to avoid what Westriech and Greenland deemed the “Table 2 Fallacy” (2). This DAG was developed in consultation with subject-matter experts in Indonesia (Bony Wiem Lestari, Kuuni Ulfah, and Panji Hadisoemarto). The DAGs depicting individual “exposure”-outcome relationships used in our modeling assumptions are found in [Figures S2b-m](#_FIGURE_3:_Directed).

The main “exposures” we were interested in studying were the effects of **provider characteristics** (age, sex, qualification, prior training on TB, and whether providers had commonly diagnosed TB patients), **facility characteristics** (facility type, whether sputum examination is available at the facility), and **visit characteristics** (length, cost, number of history-taking questions asked, number of cardinal TB symptoms asked, whether provider prescribed non-ATT antibiotic, and whether provider prescribed steroids) on the likelihood of adherence to NTP guidelines for presumptive TB management.

## Provider characteristics

Prior evidence from other SP studies on TB have indicated that provider sex (3,4), age (5), and qualification (5–8) may be related to appropriate TB management. As older providers may be more likely to be male in Indonesia, provider sex is a confounder in the relationship between age and NTP guideline adherence ([Figure S2b](#Figure3b)). SP sex is linked to provider sex in our study due to the sampling structure wherein female SPs were only assigned to visit female providers. SP sex could plausibly be related to likelihood of NTP guideline adherence if, for instance, providers know that males are more likely to have TB (9); thus, SP sex would confound the relationship between provider sex and NTP guideline adherence ([Figure S2c](#Figure3c)). In Indonesia, older providers are more likely to be specialists, specialists are more likely to be male, and specialists are more common in hospitals compared to solo practices, which would make provider age, provider sex, and facility type confounders in the relationship between provider qualification and NTP guideline adherence (10,11) ([Figure S2d](#Figure3d)). Since information on prior training on TB management was an available variable from the mapping study that had not yet been explored in prior SP studies, we were interested to see if prior training on TB management was linked to NTP guideline adherence (12). We posited that provider age could be a confounder if older providers were more likely to have received the training, or if younger providers are more likely to have received this type of training in medical school. Provider qualification could also be a confounder if specialists are more likely to receive this training compared to general practitioners ([Figure S2e](#Figure3e)). Similarly, we were curious if providers who have seen TB in their clinics would be more likely to adhere to NTP guidelines. Provider age could be a confounder here if older providers are more likely to have seen TB in their practices due to having more years of experience practicing medicine. Additionally, provider qualification could confound the relationship between having diagnosed a TB patient and NTP guideline adherence if specialists are more likely to diagnose TB patients regularly. Finally, provider receiving training on TB management could make a provider more likely to adhere to NTP guidelines, which would make it a confounder in this relationship ([Figure S2f](#Figure3f)).

## Facility characteristics

Previous SP studies have shown that the type of facility visited can affect the likelihood that an SP will receive appropriate TB care (5). Provider age is a possible confounder for the relationship between facility type and NTP guideline adherence as providers who are older and more established may be more likely to have their own solo practice ([Figure S2g](#Figure3g)). A provider may be more likely to refer a patient for sputum testing if the technology is available at their facility, so we were also interested in exploring the relationship between sputum testing availability and NTP guideline adherence. Sputum examination is more likely to be available at a hospital compared to a clinic or solo practice, which would make facility type a plausible confounder in this “exposure”-outcome relationship (13). Additionally, provider qualification may determine the likelihood that a provider works at a facility with sputum microscopy technology ([Figure S2h](#Figure3h)).

## Visit characteristics

SP studies conducted in other contexts have found relationships between visit length and appropriate management of SPs presenting with presumptive TB symptoms (3). Longer visits could imply that providers spent more time and paid more attention to an SP, or that they asked more questions, conducted more examinations, or ordered more diagnostics, which could imply stronger adherence to NTP guidelines. Number of history-taking questions would likely increase the visit length, and could itself be linked to NTP guideline adherence, therefore confounding the relationship between visit length and NTP guideline adherence ([Figure S2i](#Figure3i)). Previous studies have shown that visit cost can be negatively associated with proper management of TB cases (14). Facility type and provider qualification could be potential confounders in this relationship, as costs may be different at hospitals or clinics compared to solo practices, and specialist providers would likely charge more for their services compared to general practitioners ([Figure S2j](#Figure3j)). We were also interested in the relationship between the number of history-taking questions asked and the likelihood of NTP guideline adherence. Assuming that each of these variables are related to NTP guideline adherence, this relationship could plausibly be confounded by whether providers have received TB management training (providers who have received training on TB would be more likely to ask more history-taking questions) and provider qualification (providers with higher qualification may be more likely to ask appropriate questions) ([Figure S2k](#Figure3k)). Similarly, we hypothesized that more cardinal TB symptoms asked about – defined as cough, blood in sputum, fever, night sweats, and weight loss – could be positively associated with NTP guideline adherence; this association has been shown in other SP studies (3). Potential confounders in this relationship are the same as those in the relationship between our outcome and number of history-taking questions asked – provider received training on TB management and provider qualification – with the addition of number of history-taking questions asked, an increase in which could increase the likelihood of asking about more cardinal TB symptoms ([Figure S2l](#Figure3l)). Finally, since previous SP studies have found high rates of inappropriate prescribing of broad-spectrum non-ATT antibiotics and steroids (8,14,15), we wanted to explore if inappropriate prescribing was correlated with NTP guideline adherence in our setting. The relationship between these variables and our outcome could be confounded by provider qualification (specialists may be less likely to prescribe incorrectly) and whether the provider had received TB management training (providers who have received prior TB training could be less likely to prescribe inappropriate medications) (5–8) ([Figure S2m](#Figure3m)).

## FIGURE S2-a: Directed Acyclic Graph (DAG) depicting overall relationships among variables and individual “exposure”-outcome relationships in the cross-sectional analysis of COVET-only SP results


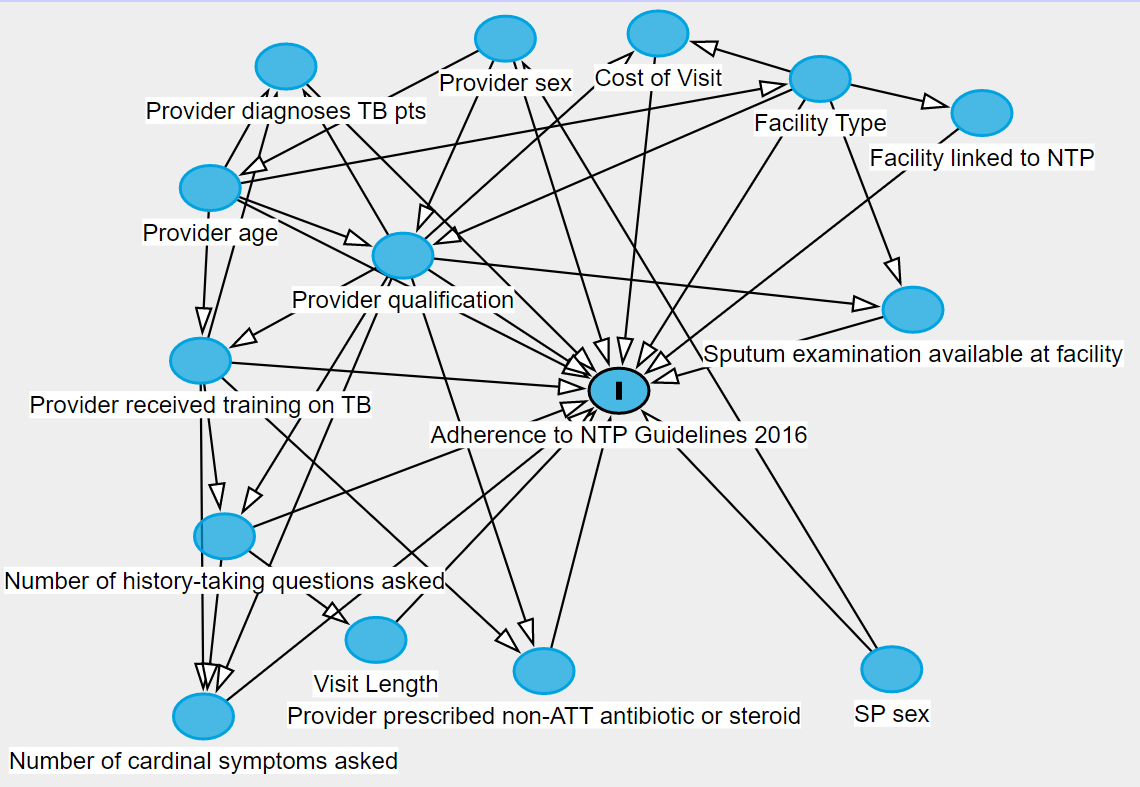


Figure S2-a: Directed acyclic graph depicting relationships between variables in the cross-sectional analysis of COVET-only SP results

| 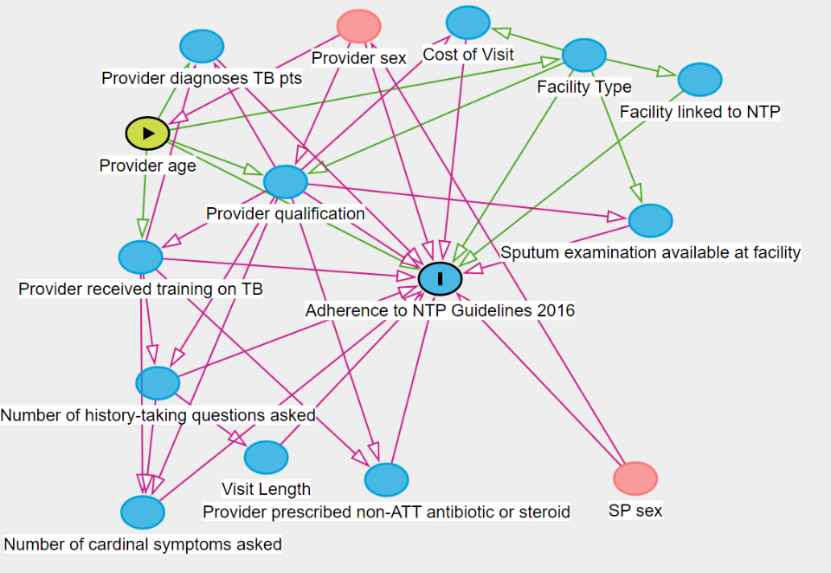  Figure S2-b Exposure = Provider age. Minimal sufficient adjustment by provider sex. | | | 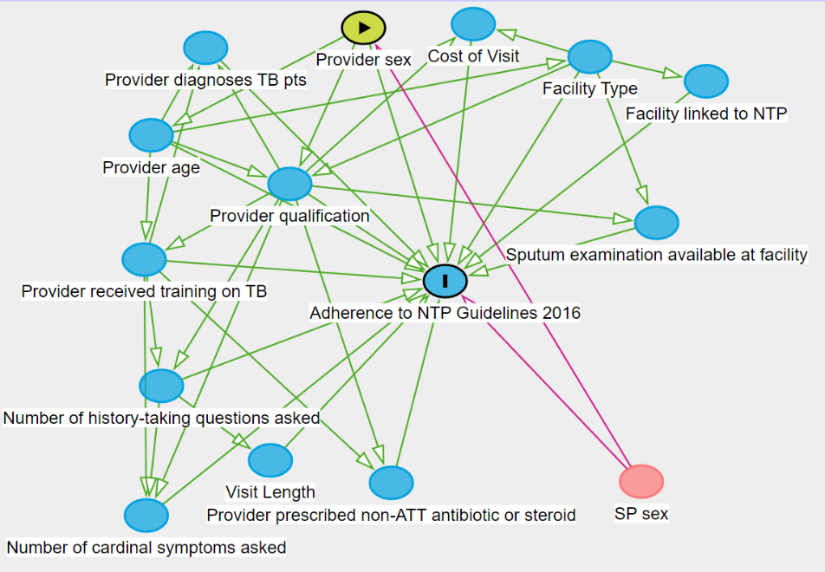  Figure S2-c Exposure = Provider sex. Minimal sufficient adjustment by SP sex. |
| --- | --- | --- | --- |
| 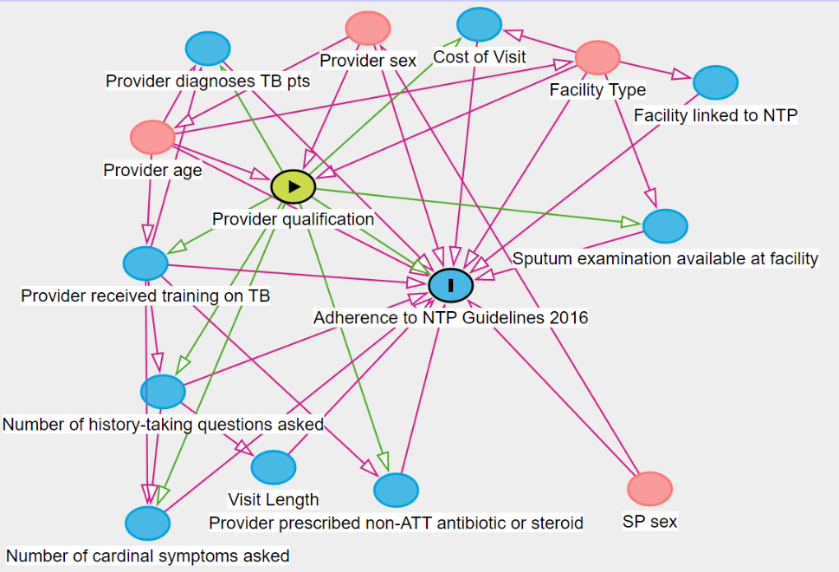  Figure S2-d Exposure = Provider qualification. Minimal sufficient adjustment by facility type, provider age, and provider sex. | | | 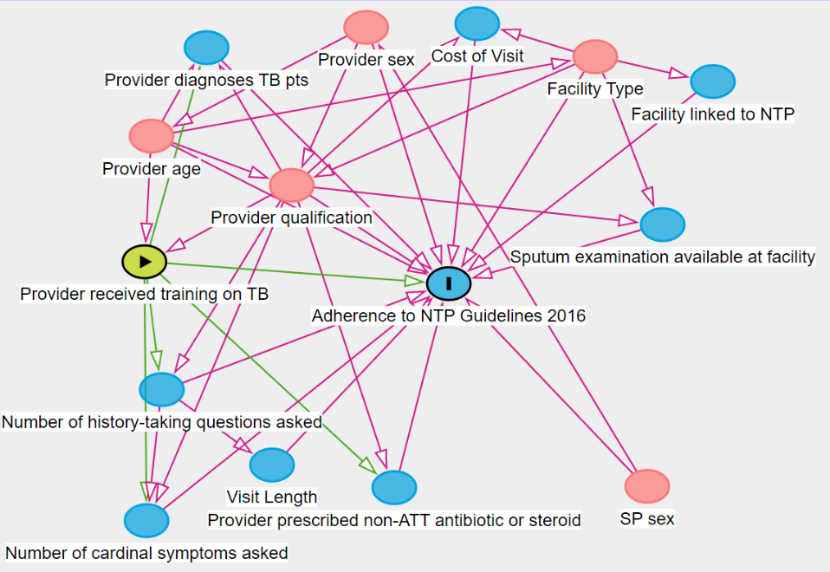  Figure S2-e Exposure = Provider received training on TB. Minimal sufficient adjustment by provider age, provider qualification, and provider diagnoses at least one TB case per month |
| 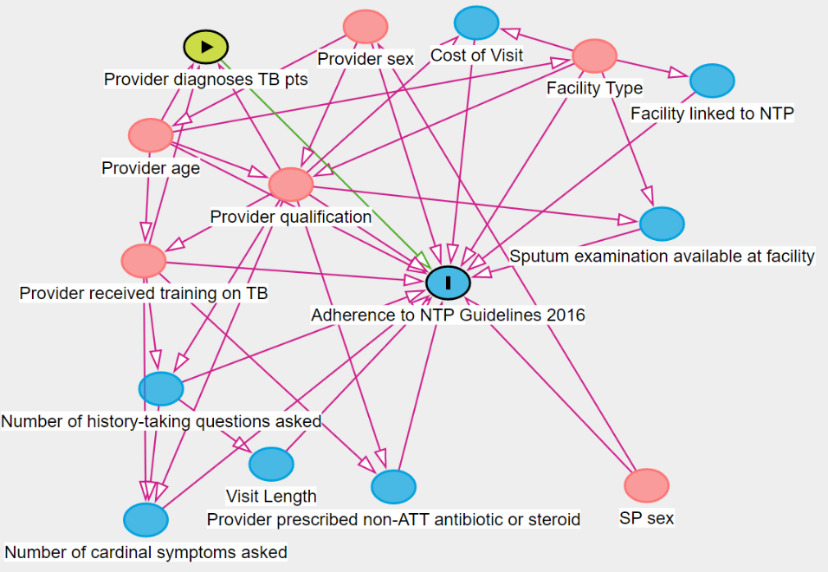  Figure S2-f Exposure = Provider diagnoses at least 1 TB case per month. Minimal sufficient adjustment by provider age, provider qualification, and provider received training on TB. | 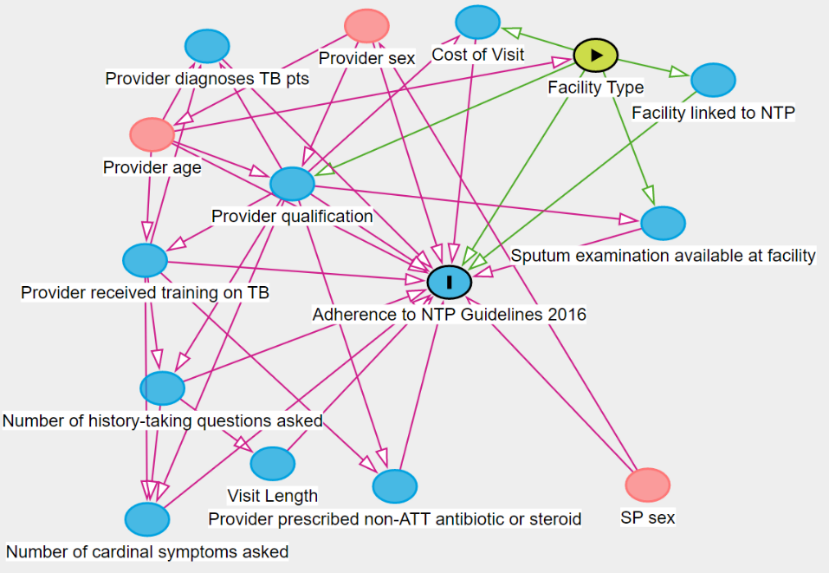  Figure S2-g Exposure = Facility type. Minimal sufficient adjustment by provider age. | | |
| 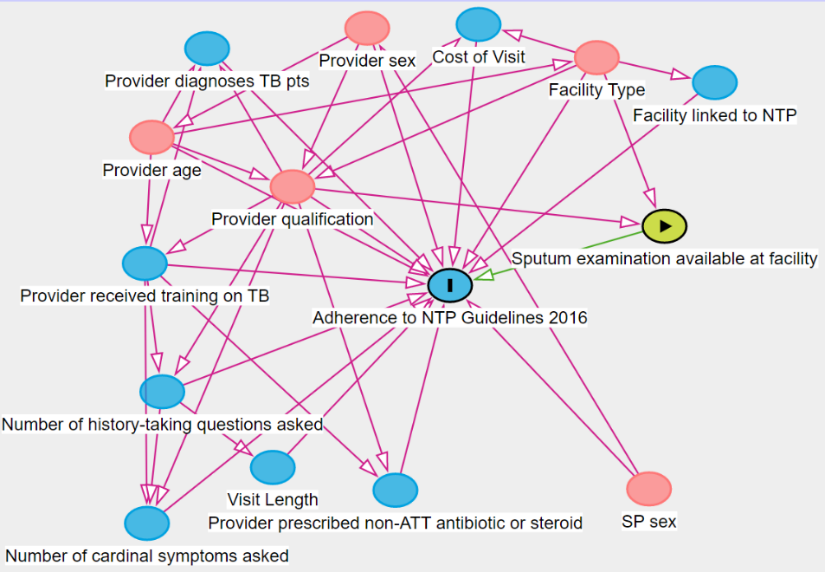  Figure S2-h Exposure = Sputum examination available at facility. Minimal sufficient adjustment by facility type and provider qualification. | 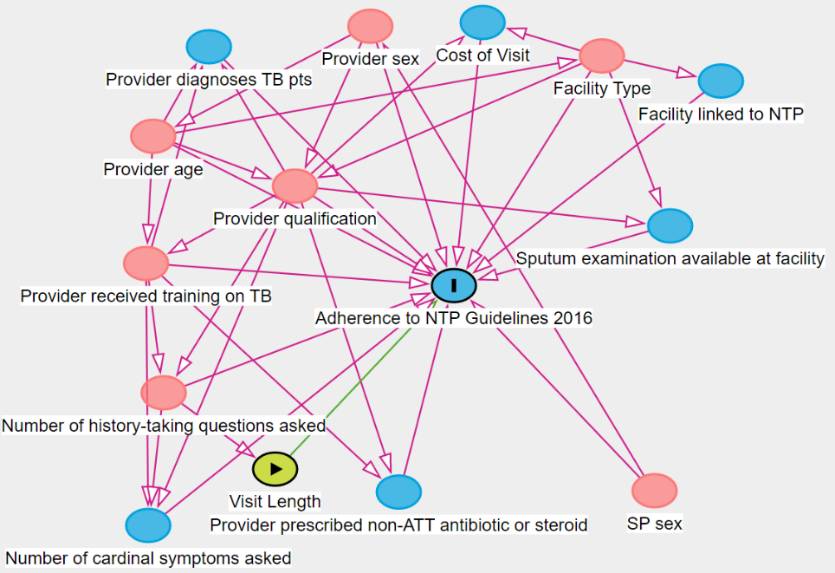  Figure S2-i Exposure = Visit length. Minimal sufficient adjustment by number of history-taking questions asked. | | |
| 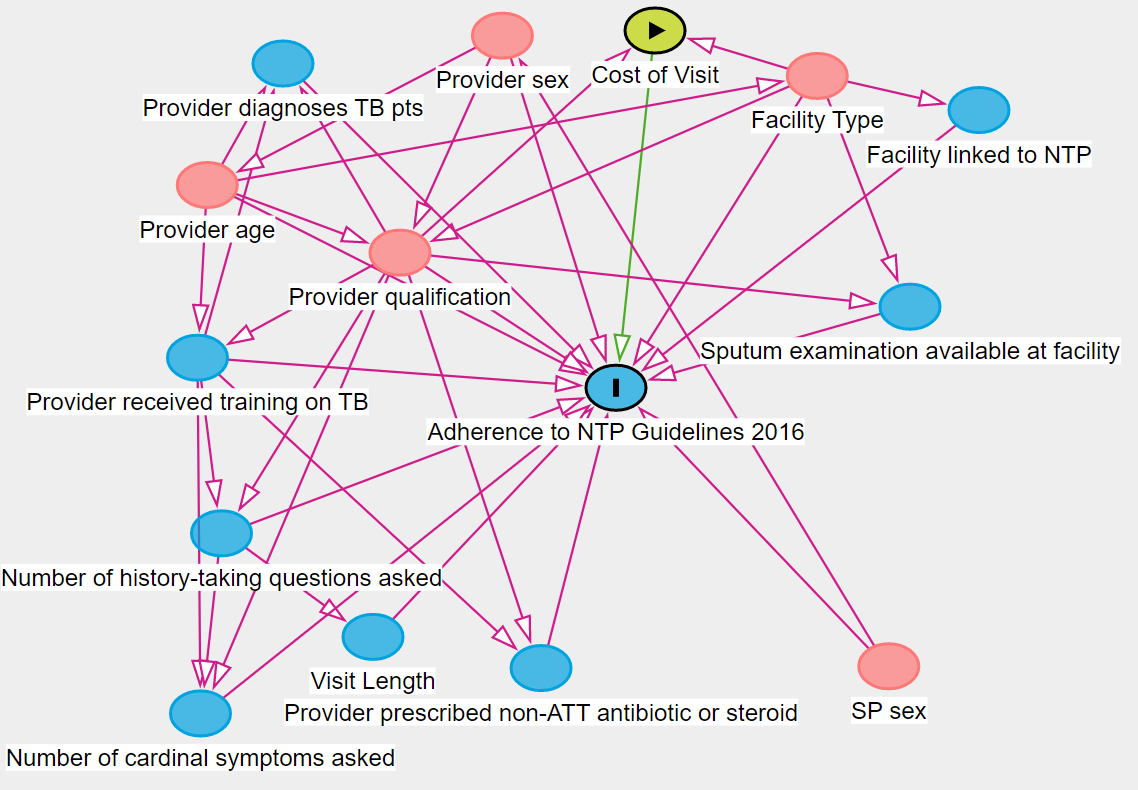  Figure S2-j Exposure = Visit cost. Minimal sufficient adjustment by facility type and provider qualification. | | 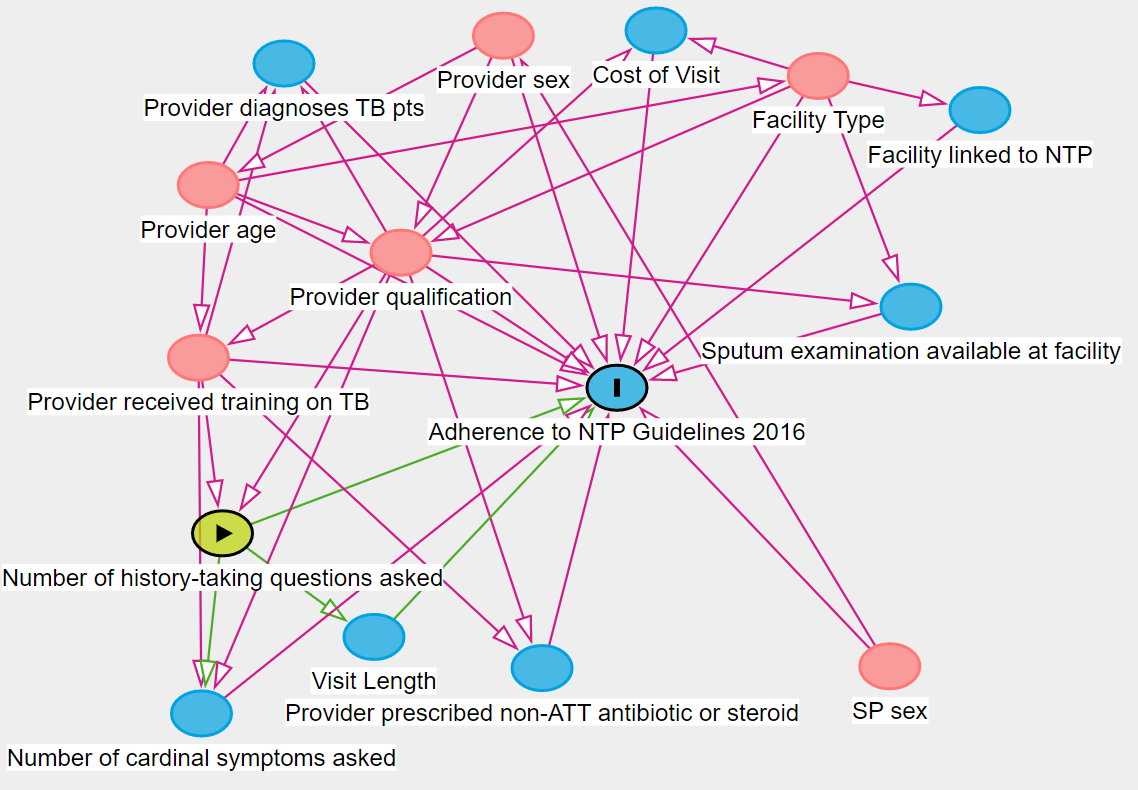  Figure S2-k Exposure = Number of history-taking questions asked. Minimal sufficient adjustment by provider qualification and provider received training on TB | |
| 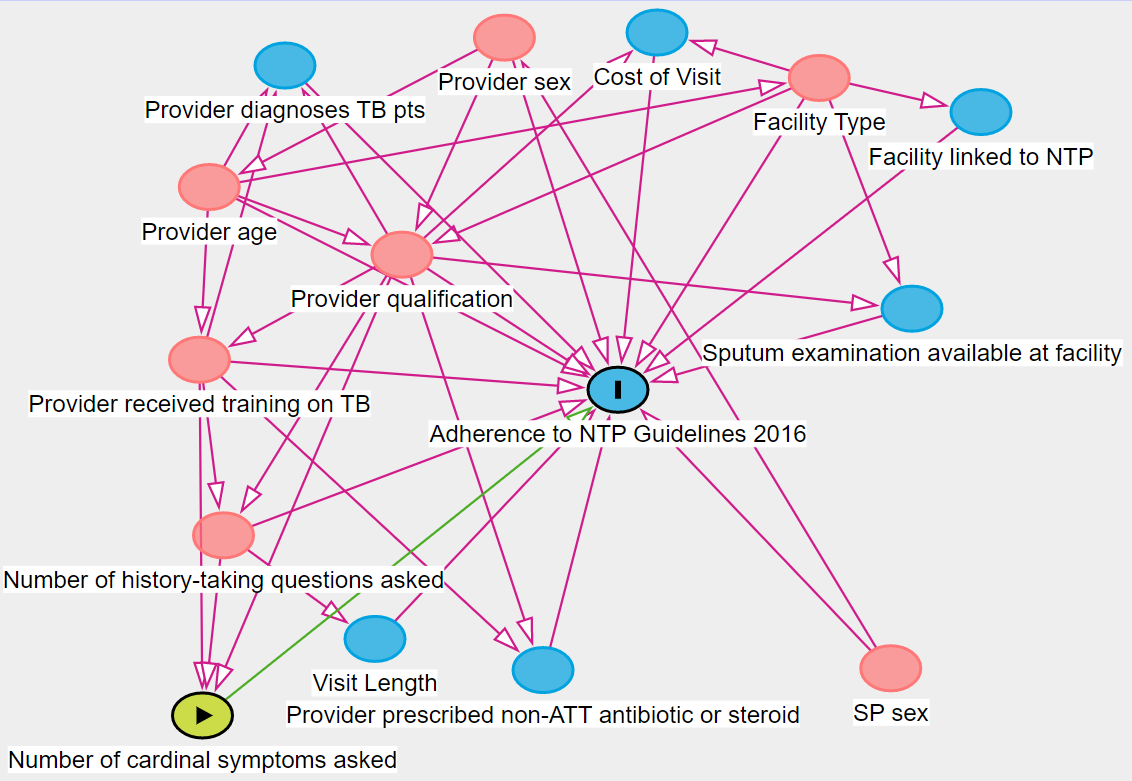  Figure S2-l Exposure = Number of cardinal TB symptoms asked. Minimal sufficient adjustment by number of history-taking questions asked, provider qualification, and provider received training on TB. | | 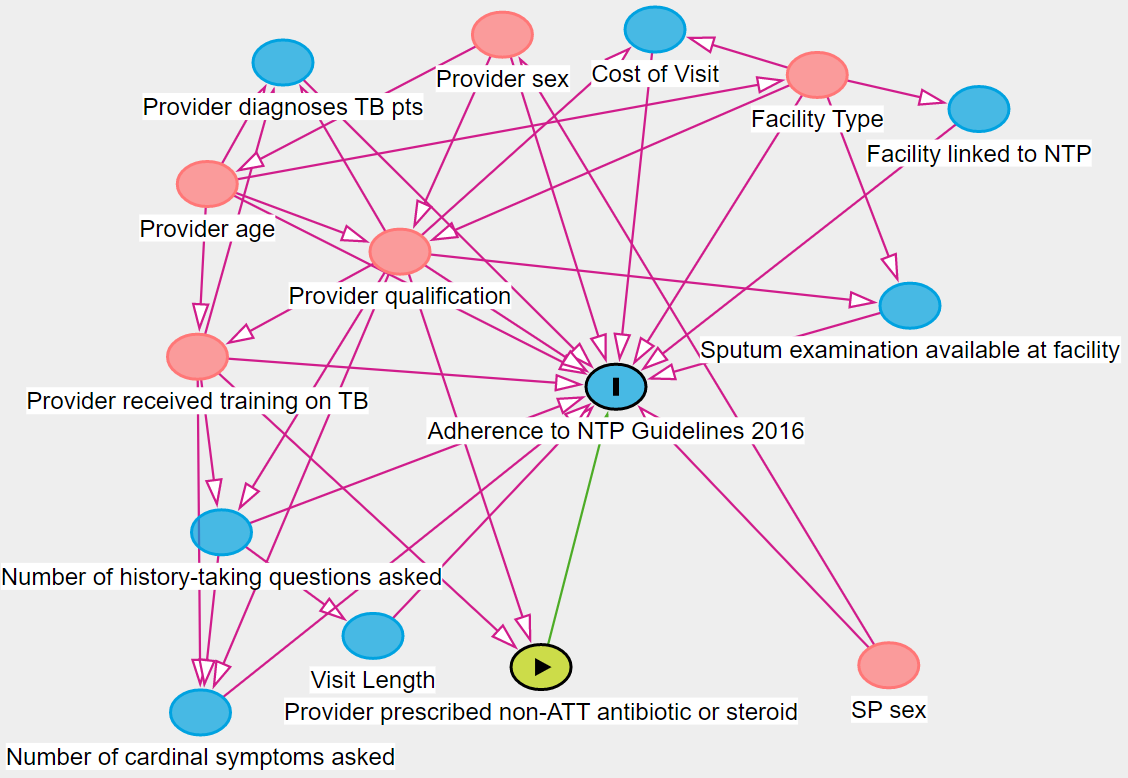  Figure S2-m Exposure = Provider prescribed non-ATT antibiotic or steroid. Minimal sufficient adjustment by provider qualification and provider received training on TB. | |

## References

1. Tennant PWG, Murray EJ, Arnold KF, Berrie L, Fox MP, Gadd SC, et al. Use of directed acyclic graphs (DAGs) to identify confounders in applied health research: review and recommendations. Int J Epidemiol. 2021 Apr 1;50(2):620–32.

2. Westreich D, Greenland S. The Table 2 Fallacy: Presenting and Interpreting Confounder and Modifier Coefficients. Am J Epidemiol. 2013 Feb 15;177(4):292–8.

3. Boffa J, Moyo S, Chikovore J, Salomon A, Daniels B, Kwan AT, et al. Quality of care for tuberculosis and HIV in the private health sector: a cross-sectional, standardised patient study in South Africa. BMJ Glob Health. 2021 May;6(5):e005250.

4. Rosapep LA, Faye S, Johns B, Olusola-Faleye B, Baruwa EM, Sorum MK, et al. Tuberculosis care quality in urban Nigeria: A cross-sectional study of adherence to screening and treatment initiation guidelines in multi-cadre networks of private health service providers. PLOS Glob Public Health. 2022 Jan 6;2(1):e0000150.

5. Sylvia S, Xue H, Zhou C, Shi Y, Yi H, Zhou H, et al. Tuberculosis detection and the challenges of integrated care in rural China: A cross-sectional standardized patient study. Evans C, editor. PLOS Med. 2017 Oct 17;14(10):e1002405.

6. Das J, Kwan A, Daniels B, Satyanarayana S, Subbaraman R, Bergkvist S, et al. Use of standardised patients to assess quality of tuberculosis care: a pilot, cross-sectional study. Lancet Infect Dis. 2015 Nov 1;15(11):1305–13.

7. Kwan A, Daniels B, Saria V, Satyanarayana S, Subbaraman R, McDowell A, et al. Variations in the quality of tuberculosis care in urban India: A cross-sectional, standardized patient study in two cities. PLoS Med. 2018 Sep;15(9):e1002653.

8. Daniels B, Shah D, Kwan AT, Das R, Das V, Puri V, et al. Tuberculosis diagnosis and management in the public versus private sector: a standardised patients study in Mumbai, India. BMJ Glob Health. 2022 Oct 1;7(10):e009657.

9. World Health Organization. Global tuberculosis report 2022 [Internet]. Geneva: World Health Organization; 2022 [cited 2022 Nov 9]. Available from: https://www.who.int/publications-detail-redirect/9789240061729

10. Kementerian Kesehatan Republik Indonesia. Indonesia Health Profile 2019 [Profil Kesehatan Indonesia Tahun 2019] [Internet]. Jakarta, Indonesia: Kementerian Kesehatan Republik Indonesia; 2020 [cited 2022 Nov 30]. Available from: https://www.kemkes.go.id/article/view/20082400001/profil-kesehatan-indonesia-2019.html

11. Meliala A, Hort K, Trisnantoro L. Addressing the unequal geographic distribution of specialist doctors in indonesia: the role of the private sector and effectiveness of current regulations. Soc Sci Med 1982. 2013 Apr;82:30–4.

12. Wu S, Roychowdhury I, Khan M. Evaluating the impact of healthcare provider training to improve tuberculosis management: a systematic review of methods and outcome indicators used. Int J Infect Dis. 2017 Mar 1;56:105–10.

13. Mahendradhata Y, Trisnantoro L, Listyadewi S, Soewondo P, Marthias T, Harimurti P, et al. The Republic of Indonesia Health System Review. World Health Organization; 2017. (Health systems in transition).

14. Xue H, Hager J, An Q, Liu K, Zhang J, Auden E, et al. The Quality of Tuberculosis Care in Urban Migrant Clinics in China. J Environ Res. 2018;15(9).

15. Salomon A, Boffa J, Moyo S, Chikovore J, Sulis G, Daniels B, et al. Prescribing practices for presumptive TB among private general practitioners in South Africa: a cross-sectional, standardised patient study. BMJ Glob Health. 2022 Jan 18;7(1):e007456.

# Supplementary Table S3: Characteristics of the SP visits related to COVID-19 (COVET study only)

| **Characteristic** | **N = 292*^1^*** | **95% CI*^2^*** |
| --- | --- | --- |
| **Symptoms and History Taking** | | |
| Provider asked about runny nose/stuffy nose | 165 (57%) | 51%, 62% |
| Provider asked about nausea and/or vomiting | 100 (34%) | 29%, 40% |
| Provider asked about sore throat | 144 (49%) | 43%, 55% |
| Provider asked about impaired smell (anosmia) | 83 (28%) | 23%, 34% |
| Provider asked about indigestion and/or diarrhea | 75 (26%) | 21%, 31% |
| Provider asked about fatigue | 61 (21%) | 16%, 26% |
| Provider asked about headache | 51 (17%) | 13%, 22% |
| Provider asked about taste disturbance (ageusia) | 44 (15%) | 11%, 20% |
| Provider asked about close contact with people who are positive for COVID-19 (eating together, staying overnight, gathering in a closed room for more than 15 minutes without a mask) | 35 (12%) | 8.6%, 16% |
| Provider asked about muscle ache | 32 (11%) | 7.7%, 15% |
| Provider asked if the SP has had a swab test (PCR/Antigen/Antibody) | 60 (21%) | 16%, 26% |
| Provider asked if the SP had gone outside the city or area with high COVID-19 case numbers (red zone) | 16 (5.5%) | 3.3%, 8.9% |
| Provider asked if the SP has a previous history of suffering from COVID-19 | 13 (4.5%) | 2.5%, 7.7% |
| **Tests/Examinations Recommended** | | |
| Any COVID-19 test | 58 (20%) | 16%, 25% |
| Antigen/Rapid antigen swab test | 41 (14%) | 10%, 19% |
| PCR swab test | 28 (9.6%) | 6.6%, 14% |
| Rapid antibody test | 1 (0.3%) | 0.02%, 2.2% |
| **COVID-19 Diagnosis and Counseling** | | |
| Provider told SP they might have COVID-19 | 40 (14%) | 10%, 18% |
| Provider identified COVID-19 as the working diagnosis | 23 (7.9%) | 5.2%, 12% |
| Provider recommended SP to take the COVID-19 test offered at this facility | 27 (9.2%) | 6.3%, 13% |
| Provider explained the importance of wearing masks | 39 (13%) | 9.8%, 18% |
| Provider explained the importance of avoiding crowds | 14 (4.8%) | 2.7%, 8.1% |
| Provider explained the follow-up actions needed to be done if COVID-19 test result is positive | 12 (4.1%) | 2.2%, 7.3% |
| *^1^* n (%)  *^2^* CI = Confidence Interval  Acronyms: COVET = COVID Effects on TB Services in the Private Sector; COVID-19 = Coronavirus disease 2019; SP = Standardized Patient; PCR = Polymerase Chain Reaction | | |
